# Atmospheric Pressure Dielectric Barrier Discharge Plasma Treatment of *Alternaria* and *Fusarium* Species: Impact on Fungal Physiology, Antifungal Sensitivity, and Biofilm Formation

**DOI:** 10.3390/molecules31142422

**Published:** 2026-07-10

**Authors:** Irena Maliszewska, Daria Nowinski, Anna Baturo-Cieśniewska

**Affiliations:** 1 Department of Organic and Medical Chemistry, Faculty of Chemistry, Wrocław University of Science and Technology, 50-371 Wrocław, Poland; daria.kocek@o2.pl; 2 Department of Microbiology and Plant Ecology, Faculty of Agriculture and Biotechnology, Bydgoszcz University of Science and Technology, 85-796 Bydgoszcz, Poland; anna.baturo-ciesnieska@pbs.edu.pl

**Keywords:** fungal pathogens, controlling, non-thermal plasma, metabolic changes, fungicides, biofilm

## Abstract

This study investigated the effects of repeated dielectric barrier discharge (DBD) plasma applications on the morphological and physiological characteristics of pathogenic *Alternaria* and *Fusarium* species. Fungi, including both culture collection strains and environmental isolates, were exposed to sublethal doses of DBD plasma. The results demonstrated that the plasma exposure time required to achieve 90% cell mortality varied significantly among microorganisms, ranging from 2 min and 39 s for *Fusarium culmorum* DSM 1094 to 5 min and 19 s for *Alternaria alternata* DSM 62010. Tolerance to oxidative stress, assessed by determining the minimum inhibitory concentration (MIC) and minimum fungicidal concentration (MFC) of hydrogen peroxide, generally decreased following repeated plasma exposure. Notably, *F. tricinctum* Ft11S–23 exhibited increased resistance to hydrogen peroxide, with MIC values doubling after fifteen plasma treatments. The MFC also increased significantly, rising from 25.5 mM to 102.0 mM. Furthermore, repeated DBD plasma applications resulted in reduced tolerance of fungi to at least one of the tested fungicides; however, exceptions were observed, including increased tolerance of *F. culmorum* to specific fungicides. The capacity for biofilm formation was modulated by plasma treatment, with some species exhibiting reduced biofilm formation while others demonstrated increased capacity, depending on the specific pathogen and frequency of plasma exposure.

## 1. Introduction

The fungi kingdom is very diverse and includes organisms classified into 120,000 described species, but it is believed that the number of species in this kingdom may be as high as 5 million [1,2]. Fungi plays an important role in the environment due to its ability to decompose organic matter. On the other hand, fungi can cause health disorders in animals and humans primarily through the metabolites (mycotoxins) they produce. The most important mycotoxins are synthesized by fungal species from the genera *Fusarium*, *Alternaria*, *Penicillium* and *Aspergillus*. Trichothecenes represent a significant category of mycotoxins produced by the genus *Fusarium*, particularly concerning food and feed safety. Notably, the highly toxic T-2 and HT-2 toxins are classified as type A trichothecenes. In contrast, deoxynivalenol (DON), a type B trichothecene, is less toxic but is frequently detected in food products. Exposure to DON is associated with adverse health effects, including emesis, damage to the gastrointestinal epithelium, immune system dysregulation, and inhibition of protein synthesis [3,4]. These pathogens are also responsible for causing respiratory infections, keratitis, and invasive fusariosis, which can reach the central nervous system and other organs via hematogenous dissemination, with a mortality rate of 43–67%, especially when caused by species belonging to the *F. solani* complex [5,6].

In recent years, *Alternaria* spp. have received increasing attention in the context of the formation of mycotoxins such as AOH, AME and TeA. The first two are the most dangerous due to their carcinogenic, cytotoxic, genotoxic, and reproductive toxicity effects [7,8,9]. This genus is also a well-known biological contaminant and a very common source of potent airborne allergens. The allergen profile released from the tissues of these fungi is still poorly understood, but at least 11 allergens are currently known, including the most well-known glycoprotein Alt a1, which causes sensitization in 80% of patients. This protein is strongly associated with the occurrence and severity of asthma.

It should be noted that the increasing emergence of antifungal-resistant strains due to their widespread use is concerning and may lead to major social issues from fungal infections. Therefore, it seems justified to direct scientists’ attention to control the spread of fungi. Currently, various combinations of strategies are being proposed for fungal control, such as fungi as biocontrol agents, mycotoxin-resistant varieties, antibody-based technologies, and even nanomaterials with antifungal or mycotoxin-inhibiting activity [10]. Additionally, physical and thermal methods can be used, such as cleaning and aggressive sorting, as well as non-ionizing/ionizing irradiation [10,11]. Other methods for removing fungal and mycotoxin contamination have also been investigated, including the use of ozone, which inhibits fungal growth, and pulsed light, radio waves, and microwaves to reduce mycotoxin contamination [10]. One of the effective techniques for destroying fungi is non-thermal plasma, also called cold plasma. Plasma can effectively reduce not only pathogenic fungi, but also bacteria and viruses, and can also degrade toxins, allergens, and pesticides [12,13,14].

The exact mechanisms of microorganism inactivation using cold plasma are still debated [15]. However, numerous studies provide evidence of the biocidal properties of individual plasma components [12,16,17,18]. Reactive oxygen and nitrogen species induce oxidative stress, leading to damage to microbial cell membranes, destabilization of DNA structure and enzymes, and inactivation of proteins crucial for cell survival, leading to cell destruction or death [12,16]. Simultaneously, ions, electrons, and radicals generated in the plasma accumulate at the cell surface, generating local electrostatic stress. When electrostatic stress exceeds the tensile strength of the cell membrane, it perforates, allowing uncontrolled transport of substances into and out of the cells [17,18]. Additionally, UV photons emitted by cold plasma possess photocatalytic properties that damage cellular structures and genetic material. It was believed that the synergistic action of all plasma components enhances their properties, providing a biocidal effect [19].

Conversely, from the perspective of ecological safety associated with this technique, it is crucial to understand the changes occurring in fungal cells that may be induced by plasma exposure and this consideration is relevant for microorganisms that have survived this environmental stress. Any alterations in the cells may be preserved and could lead to the uncontrolled spread of strains with increased tolerance to factors such as oxidative stress. Currently, in this field, regarding cold plasma, the availability of literature is limited. For instance, Šimončicová et al. [20] observed morphological alterations in the hyphae of *Aspergillus flavus* treated with cold plasma, which exhibited significant structural modifications. Scanning electron microscopy revealed desiccation of the hyphae, leading to the formation of folds on their surfaces. Attenuated total reflectance Fourier-transform infrared (ATR-FTIR) spectroscopy indicated an increase in signal intensity for C=O and C-O stretching vibrations, likely associated with chemical changes in the molecular structures present on the hyphal surface. Plasma treatment resulted in elevated intracellular levels of reactive oxygen species (ROS) and a corresponding increase in the activity of antioxidant enzymes.

Our previous studies have demonstrated that atmospheric pressure dielectric barrier discharge (DBD) plasma is an effective method for eradicating filamentous fungi belonging to the genera *Alternaria*, *Botrytis*, and *Fusarium*. Additionally, sublethal doses of this plasma have been shown to induce physiological changes in these microorganisms, particularly affecting biomass production and the synthesis of cell wall-degrading enzymes (CWDEs). It was also observed that repeated plasma exposure influences the ability of fungi to infect cucumber seeds, subsequently impacting seedling development. Furthermore, we conducted studies to evaluate the capacity of phytopathogenic fungi, subjected to repeated plasma exposure, to infect tomato fruit utilizing an in vivo assay. Importantly, our findings indicated that repeated exposure of fungi to DBD plasma did not result in the development of tolerance to this treatment, as the time required to achieve 90% cell mortality remained consistent even after fifteen plasma applications [21].

This study extends our research on the control of fungal populations from the genera *Alternaria* and *Fusarium* using DBD plasma technology. This work underscores the importance of evaluating the impact of multiple cold plasma applications on the morphological and physiological changes of the examined fungi, including their oxidative stress tolerance, sensitivity to commercially available antifungal agents, as well as their capacity to form biofilms. In addition to the collection strains, our study includes the wild microorganisms *Alternaria alternata* Aa10S–23 and *Fusarium tricinctum* Ft11S–23, which were isolated from the surface of wooden vegetable storage boxes.

## 2. Results

### 2.1. Fungal Isolation

**Figure 1:**
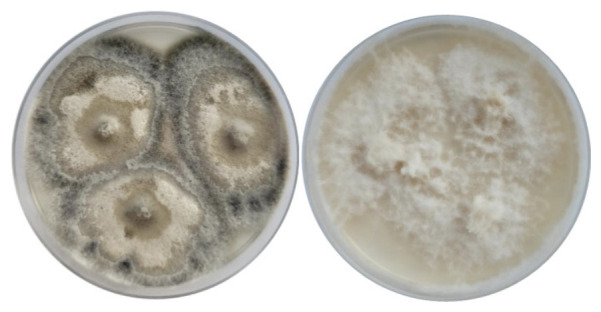
Colonies of fungi: *Alternaria alternata* Aa10S–23 (**left**) and *Fusarium tricinctum* Ft11S–23 (**right**) after 11 days of incubation on PDA medium. Scale bar (3 cm) ⟷.

Our study utilized three reference strains—*Alternaria alternata* DSM 62010, *Fusarium culmorum* DSM 1094, and *Fusarium oxysporum* DSM 12646—as well as two wild strains isolated from the surfaces of wooden vegetable storage boxes. Preliminary characterization of the isolates based on morphological features—including pigmentation, shape, elevation, colony margins, and characteristics of spores and conidiophores—enabled the classification of these fungi into the genera *Alternaria* and *Fusarium*. Nonspecific reactions demonstrated that the *Alternaria* sp. isolates were consistent with *A. alternata*, *A. arborescens*, *A. longipes*, and *A. brassicae*, while the *Fusarium* sp. isolates aligned with *F. acuminatum*, *F. tricinctum*, and *F. avenaceum*. Reactions with specific primers confirmed that the *Alternaria* sp. isolate belonged to the species *A. alternata*, and the *Fusarium* sp. isolate was identified as *F. tricinctum* (Figure 1). The ITS sequences of *A. alternata* (GenBank: PP869429.1) and *F. tricinctum* (GenBank: PP869455.1) have been deposited in the National Center for Biotechnology Information (NCBI) GenBank database.

### 2.2. Evaluation of the Effectiveness of DBD Plasma in Inactivation of Fungal Cells

A critical parameter in our experiments was the decimal reduction time (δ), defined as the duration of DBD plasma exposure required to eliminate 90% of the cells. This duration was regarded as the sublethal plasma “dose” and was utilized in all experiments involving the repeated exposure of fungi to plasma (Table 1). This parameter concerning *Fusarium oxysporum* DSM 12646, *Fusarium culmorum* DSM 1094, and *Alternaria alternata* DSM 62010 was presented in our previous work [21], while the values of δ related to *Fusarium tricinctum* Ft11S–23f and *Alternaria alternata* Aa10S–23 were determined experimentally in the current study.

Determination of the *δ* value revealed differences in the sensitivity of the tested microorganisms to DBD plasma. *F. culmorum* DSM 1094 exhibited the highest sensitivity, followed by *F. oxysporum* DSM 12646, *F. tricinctum* Ft11S–23, *A. alternata* Aa10S–23, and *A. alternata* DSM 62010.

### 2.3. Effect of Multiple DBD Plasma Treatments on Morphological and Physiological Changes in the Studied Fungi

**Figure 2:**
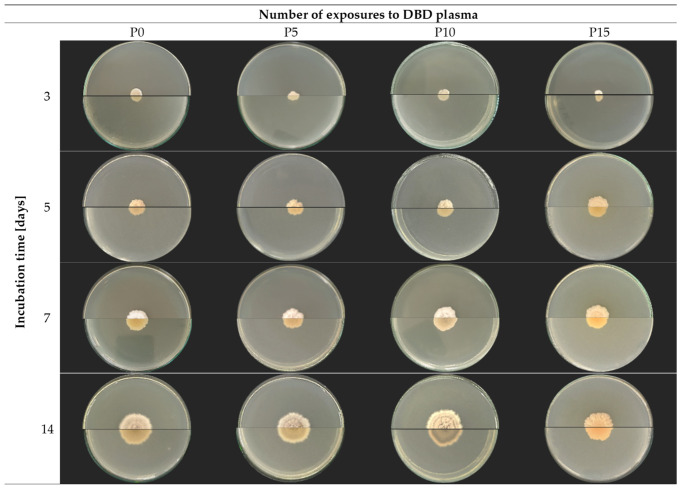
The morphology of *A. alternata* DSM 62010 colonies was assessed in untreated samples (P0) and after five (P5), ten (P10), and fifteen (P15) DBD plasma treatments on potato dextrose agar (PDA) over a 14-day culture period. The upper portion of each image displays the colony’s obverse view, while the lower portion presents the reverse view. Images were captured after 3, 5, 7, and 14 days of incubation. Scale bar (3 cm) ⟷.

**Figure 3:**
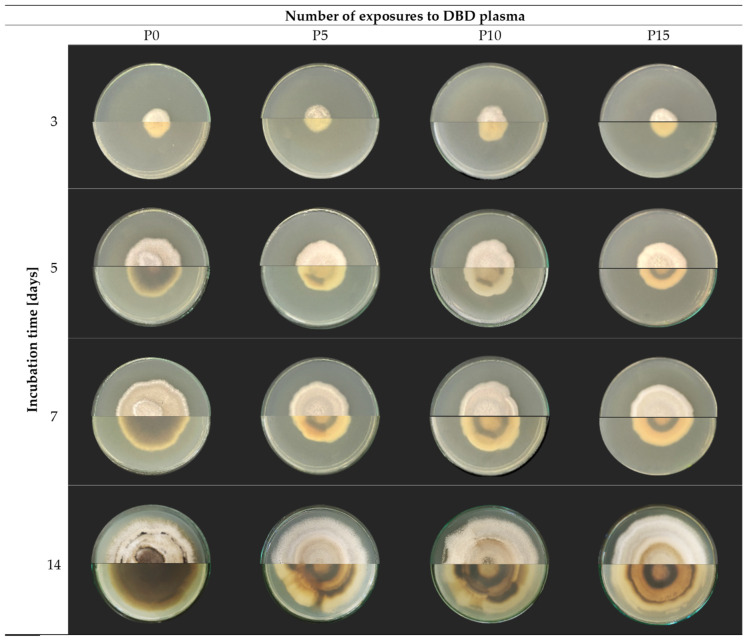
The morphology of *A. alternata* Aa10S–23 colonies was assessed in untreated samples (P0) and after five (P5), ten (P10), and fifteen (P15) DBD plasma treatments on potato dextrose agar (PDA) over a 14-day culture period. The upper portion of each image displays the colony’s obverse view, while the lower portion presents the reverse view. Images were captured after 3, 5, 7, and 14 days of incubation. Scale bar (3 cm) ⟷.

The impact of multiple DBD plasma treatment on the morphological changes in the mycelium of five tested fungal strains was evaluated using a sublethal plasma dose, which was individually determined for each microorganism (see Table 1). The application of a sublethal dose allowed for the survival of a sufficiently large number of microorganisms, facilitating the observation of morphological changes induced by exposure to the biocidal agent. Macroscopic assessments of fungal growth on the PDA medium were conducted for both untreated samples (P0) and those subjected to five (P5), ten (P10), and fifteen (P15) exposures to the sublethal plasma dose. Changes in colony morphology following multiple DBD plasma treatment are shown in Figure 2 and Figure 3 and Figures S1–S3 (Supplementary Information).

Figure 2 illustrates the morphological changes in colonies formed by *A. alternata* DSM 62010 due to multiple exposures to DBD plasma. Notably, *A. alternata* DSM 62010 exhibited limited growth on PDA medium over the 14-day experimental period. The shape of the colonies was irregular with filamentous margins. A difference in pigmentation was evident among the colonies formed by mycelia P0, P5, P10, and P15, with colonies from P15 exhibiting a brownish-orange color, while the others appeared light brown. For comparison, the morphological changes in colonies formed by the wild strain of *A. alternata* Aa10S-23 as induced by DBD plasma are illustrated in Figure 3. As can be seen, this fungi produced colonies with wavy margins and uneven pigmentation. The aerial mycelium appeared beige-brown, while the reverse side of colony exhibited brown (P0) or brownish-orange (P5, P10, P15) coloration.

The morphological changes in colonies induced by multiple exposure of the tested *Fusarium* strains to plasma are presented in Figures S1–S3. Colonies of the fungus *F*. *oxysporum* DSM 12646 (Figure S1) that were not treated with plasma (P0) and those subjected to multiple treatments (P5, P10, P15) developed white aerial mycelium with a pinkish-yellow coloration visible on the reverse side. Colonies P0 and P10 exhibited wavy margins, in contrast to the smooth margins of colonies P5 and P15. For *F. culmorum* DSM 1094, a significant difference in colony size was observed between the untreated mycelium (P0) and that subjected to multiple plasma treatments (P5, P10, P15) (Figure S2). The colonies formed by *F. culmorum* DSM 1094 displayed white mycelium, which turned light pink with prolonged incubation on PDA medium.

Figure S3 depicted the morphological changes in colonies of wild strain isolated from the surfaces of wooden storage crates. *F. tricinctum* Ft11S–23 initially formed white mycelium, which adopted a light pink coloration over time on solid medium. Colonies formed after multiple treatments of *F. tricinctum* Ft11S–23 with cold plasma (P5, P10, P15) exhibited significantly smaller sizes compared to the control (P0), as well as irregular shapes and dense aerial mycelium. A yellow coloration was also visible on the reverse side of the colonies.

Measurements of colony diameter were also taken during the cultivation of the fungi on solid media. The results are presented in Table S1 (Supplementary Information). Colonies formed by *F. oxysporum* DSM 12646 and *A. alternata* DSM 62010 subjected to multiple exposures to DBD plasma did not show significant differences in size during cultivation on solid media compared to the control (P0). In contrast, the mycelium of *F. tricinctum* Ft11S–23 exhibited a significantly slower growth rate on PDA medium following multiple exposures to DBD plasma (P5, P10, P15) compared to the control (P0). After 3 days of incubation, the diameters of colonies designated as P5, P10, and P15 were approximately 25% smaller than P0 (2.7 cm ± 0.1). After 5 and 7 days of incubation, this difference increased to approximately 48%, while after 14 days, the diameters of colonies P5, P10, and P15 were approximately 33%, 22%, and 25% smaller than the control (P0), respectively.

Conversely, the mycelium of *F. culmorum* DSM 1094 exhibited significantly faster growth after the fifth (P5), tenth (P10), and fifteenth (P15) plasma treatments compared to the untreated mycelium (P0). The diameters of colonies P5, P10, and P15 were 179%, 187%, and 205% larger than P0 (1.0 ± 0.1 cm) after 3 days of incubation. After 5 and 7 days of incubation, the differences in colony size between P5, P10, P15, and P0 averaged 228%, and by the end of the test, the diameters of colonies formed by *F. culmorum* DSM 1094 after multiple plasma exposures were approximately 81–85% larger than the control (P0).

A stimulating effect of plasma on the growth of *A. alternata* Aa10S–23 on PDA medium was also observed (Figure 3). However, the differences in colony size between mycelia subjected to multiple plasma exposures and the control were significantly smaller compared to *F. culmorum* DSM 1094, not exceeding 10% after 3, 5, and 7 days of incubation. By the end of the experiment, the diameters of colonies formed by mycelia P5, P10, and P15 were approximately 11%, 23%, and 27% larger compared to P0 (6.2 ± 1.7 cm).

### 2.4. Oxidative Stress Tolerance in the Studied Fungi Subjected to Multiple Exposures to DBD Plasma

The effect of multiple treatments with a sublethal dose of DBD plasma on the oxidative stress tolerance of the tested filamentous fungi was investigated using hydrogen peroxide (H_2_O_2_) as an inducer of oxidative stress. In the initial phase of the experiments assessing the tolerance of the tested fungi to oxidative stress, the minimum inhibitory concentration (MIC) and minimum fungicidal concentration (MFC) values for H_2_O_2_ were determined, and the results are summarized in Table 2. MIC values were determined based on visual assessment of fungal growth in liquid media containing H_2_O_2_, while MFC values were assessed by evaluating colony growth after 24 h of incubation in the presence of H_2_O_2_, followed by sub-culturing onto PDA medium without the stressor. The results obtained suggested that repeated treatment of fungi with DBD plasma may alter the sensitivity of these microorganisms to H_2_O_2_. Analysis of these results indicates that while changes in MIC and MFC values are not significant from a practical standpoint, they should not be overlooked.

For example, the MIC of H_2_O_2_ against *F. oxysporum* DSM 12646 remained unchanged after plasma exposures (12.8 mM); however, the MFC decreased twofold compared to P0 (to 25.5 mM) after fifteen plasma expositions (P15). A twofold decrease in the MIC values and a fourfold decrease in the MFC of hydrogen peroxide against *F. culmorum* DSM 1094 were also observed as a result of plasma treatments (P5, P10, P15). For *A. alternata* DSM 62010 and *A. alternata* Aa10S–23, a twofold reduction in MIC values was noted after the tenth (P10) and fifteenth (P15) plasma doses. Specifically, for *A. alternata* DSM 62010 mycelia, a twofold reduction in MIC values was observed after both the tenth (P10) and fifteenth (P15) plasma doses, while for the *A. alternata* Aa10S–23, a twofold reduction in MIC values was observed after fifteen plasma treatments (P15) compared to the control (P0).

An exception to the observed decreased sensitivity to hydrogen peroxide was noted for the *Fusarium tricinctum* Ft11S–23 strain, for which the MIC doubled after fifteen plasma treatments (P15). Meanwhile, the MFC increased progressively with increasing plasma exposure, rising from 25.5 mM for P0 to 102.0 mM for P15.

Based on the obtained results, it is reasonable to correlate the growth rate of fungi (as measured by colony diameter) with their sensitivity to the cytotoxic effects of hydrogen peroxide. This correlation is supported by the findings for *F. culmorum* DSM 1094, whose mycelium not treated with DBD plasma (P0) exhibited significantly slower growth on solid medium (with a colony diameter of 4.5 ± 0.1 cm after 14 days of incubation) compared to mycelium subjected to multiple treatments (with colony diameters for P5, P10, and P15 ranging from 8.1 cm to 8.3 cm), while also demonstrating the lowest sensitivity to H_2_O_2_. Similarly, for *F. tricinctum* Ft11S–23, the largest colony diameter (8.1 ± 0.1 cm) and the highest sensitivity to oxidative stress were observed in the untreated mycelium (P0). However, following repeated plasma treatments, a decrease in colony growth rate was noted (with colony diameters for P5, P10, and P15 ranging from 5.4 cm to 6.3 cm) alongside an increase in tolerance to H_2_O_2_.

In contrast, *A. alternata* Aa10S–23 exhibited the fastest growth on solid media after ten (P10) and fifteen (P15) DBD plasma treatments, with colony diameters after 14 days of incubation being approximately 23% and 27% larger, respectively, compared to P0, while simultaneously showing increased sensitivity to H_2_O_2_.

**Figure 4:**
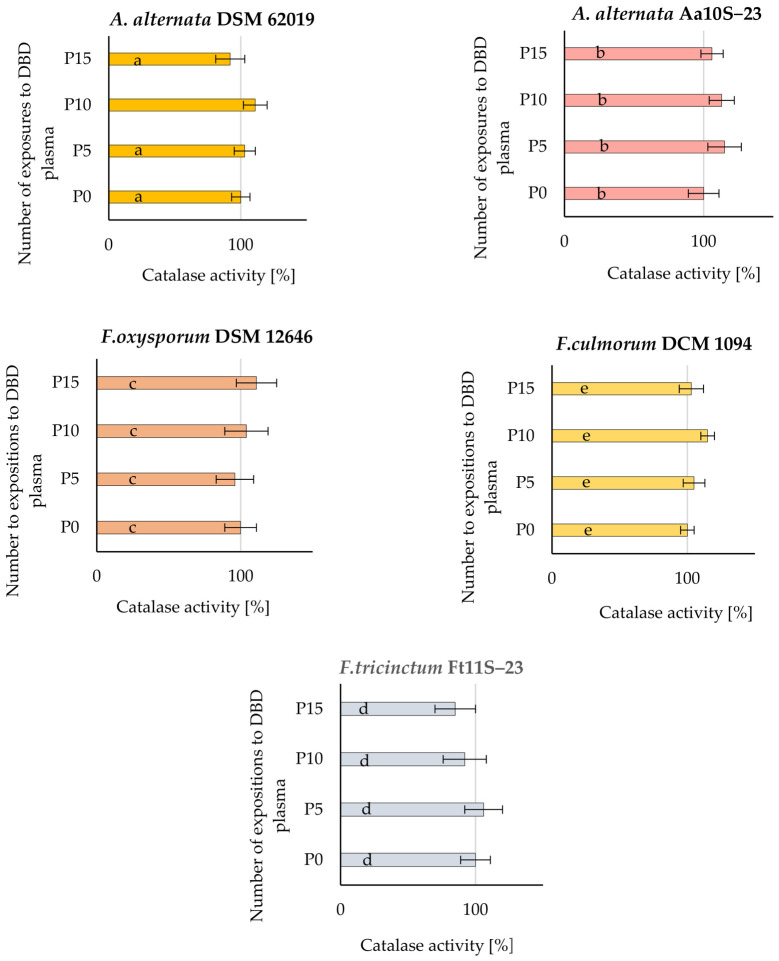
Effect of multiple DBD plasma exposures on the catalase activity of the studied fungi. Data are presented as the mean ± standard deviation of six independent replicates (*n* = 6). Relative catalase activity was calculated by normalizing the values to the untreated control (P0), which was defined as 100%. a, b, c, d, and e indicate significant differences according to Dunnett’s post hoc test following one-way ANOVA (*p* < 0.05).

In the second part of our experiments examining changes in fungal tolerance to oxidative stress induced by repeated exposure to DBD plasma, the activity of catalase expressed in cells of the studied molds was measured after pre-incubation with hydrogen peroxide (Figure 4).

The results obtained suggest that the level of this enzyme in cells is influenced by the number of mold exposures to DBD plasma. However, this relationship was not consistent and varied depending on both the strain tested and the number of plasma exposures. In some cases, catalase activity in cells subjected to repeated plasma treatment was increased, while in others, it was decreased compared to the P0 controls. For example, in *A. alternata* Aa10S–23 and *F. culmorum* DCM 1094, catalase activity in cells subjected to 5 and 10 plasma treatments (P5, P10) increased by approximately 13–15% compared to untreated control cells (P0). In *F. tricinctum* Ft115–23 cells, catalase activity exhibited an initial increase of approximately 6% following five plasma treatments (P5). However, subsequent exposures resulted in a decrease in catalase activity, with reductions of 8–15% observed after ten (P10) and fifteen (P15) treatments. These findings collectively demonstrate that DBD plasma modulates catalase activity in a manner that is dependent on the number of cell exposures and is also strain-specific.

### 2.5. Effect of Multiple DBD Plasma Treatment of Fungi on Changes in Some Pathogenicity Factors

#### 2.5.1. Alterations in the Sensitivity of Fungi Subjected to Multiple Plasma Exposures to Fungicides

The effect of multiple sublethal doses of DBD plasma on the sensitivity of the tested fungi to six different commercially available fungicides (F1–F6) is shown in Table 3 and Table 4. Untreated cells (P0) served as controls. The results are based on visual assessment of mycelial growth in the presence of various concentrations of the fungicide on a liquid medium (MIC values) or on a solid medium (MFC values). Table 3 lists the determined MIC and MFC values of fungicides F1–F3 in relation to the tested fungi. For these products, the MIC and MFC values were expressed in µL mL^−1^ because the tested biocides were commercially available in liquid form (emulsion).

Table 4 presents the MIC and MFC values determined for the F4–F6 fungicides. Since these fungicidal products were in powder form, the MIC and MFC values are expressed in mg mL^−1^. The analysis of fungal sensitivity for fungicides F1–F6 was conducted within the concentration range recommended by the manufacturer for practical use (see Section 4.8; Table 5).

In the case of *A. alternata* DSM 62010 (Table 3 and Table 4), tolerance changes were detected concerning most of the antifungal agents tested following multiple exposures to DBD plasma, with the exception of F3. The MIC and MFC values for F1 gradually decreased from 40 µL mL^−1^ and >40 µL mL^−1^ for plasma-untreated mycelium (P0) to 2.5 µL mL^−1^ and 20 µL mL^−1^ for mycelium P15 (Table 3). In the case of F2, increased sensitivity was observed only after fifteen plasma expositions (P15), for which the MFC was twofold lower than for the others (7.81 × 10^−2^ µL mL^−1^) (Table 3). A decrease in MFC values against F4 was also noted for mycelium P10 (5.0 mg mL^−1^) and P15 (2.5 mg mL^−1^) compared to mycelium P0 (>40 mgmL^−1^), with no change in MIC (2.5 mg mL^−1^) (Table 4).

Tolerance increased only for F6 (Table 4), for which the minimum inhibitory concentration (MIC) and minimum fungicidal concentration (MFC) for plasma-untreated mycelium (P0) were below the measurable range (<4.88 × 10^−3^ mg mL^−1^), while the MFC values increased at least twofold (to 9.77 × 10^−3^ mg mL^−1^) after plasma treatment (P5, P10, P15).

For *Alternaria alternata* Aa10S–23, no changes in sensitivity were observed for F2, F3, and F5 (Table 3 and Table 4). Increased sensitivity to F1 appeared after fifteen plasma expositions (Table 3). Conversely, for F4 and F6, a gradual increase in sensitivity was observed with an increasing number of dielectric barrier discharge plasma treatments (Table 4).

Multiple DBD plasma treatments of *F. oxysporum* DSM 12646 resulted in increased susceptibility to the fungicides designated as F1, F2, and F4 (Table 3 and Table 4). Specifically, the minimum inhibitory concentration (MIC) for F1 decreased from 40 µL mL^−1^ in untreated mycelium (P0) to 10 µL mL^−1^ after repeated plasma treatments (P5, P15) (Table 3). However, no significant change was observed in the minimum fungicidal concentration (MFC), which remained above 40 µL mL^−1^. An increase in the sensitivity of P10 and P15 mycelium relative to F2 was also noted.

When F4 was used in the experiments (Table 4), the MFC value for this strain decreased from >40 mg mL^−1^ (P0) to 10 mg mL^−1^ after repeated plasma treatment, without changing the MIC value (2.5 mg mL^−1^). Tolerance to fungicide F6 increased, with MIC and MFC values rising from 3.91 × 10^−2^ mg mL^−1^ and >40 mg mL^−1^ in untreated mycelia (P0) to 7.81 × 10^−2^ mg mL^−1^ and 0.313 mg mL^−1^ in P15, respectively (Table 4). No significant changes in sensitivity were detected for fungicides F3 and F5.

Multiple treatments of *F. culmorum* DSM 1094 mycelium with sublethal doses of DBD plasma affected its sensitivity to most tested fungicides, except F1. Tolerance increased for F2 and F3 (Table 3), whereas tolerance decreased for F4, F5, and F6 (Table 4). The MIC and MFC values for F2 increased progressively from below 4.88 × 10^−3^ µL mL^−1^ and 1.56 × 10^−1^ µL mL^−1^ in untreated mycelia (P0) to 3.13 × 10^−1^ µL mL^−1^ after 15 treatments (P15). Notably, significant changes in susceptibility were observed for F3: untreated mycelia (P0) exhibited high sensitivity (MIC and MFC < 6.25 × 10^−1^ µL mL^−1^), whereas repeatedly treated mycelia (P5, P10, P15) showed increased MIC and MFC values, reaching 20 µL mL^−1^ and >40 µL mL^−1^, respectively. Conversely, repeated plasma treatments resulted in increased sensitivity to fungicides F4, F5, and F6, as evidenced by decreased MIC and MFC values relative to the control (Table 4). For fungicides F4, MIC values fell below the detection limit (<6.25 × 10^−1^ mg mL^−1^), and MFC values decreased fourfold after plasma exposure. For F5, MIC and MFC values decreased from 40 mg mL^−1^ and >40 mg mL^−1^ in P0 to below 6.25 × 10^−1^ mg mL^−1^ and 2.5 mg mL^−1^ in P15. Similarly, for F6, initial MIC and MFC values decreased significantly after five plasma treatments, with stabilization observed after ten (P10) and fifteen (P15) treatments at approximately 3.91 × 10^−2^ mg mL^−1^.

In *F. tricinctum* Ft11S–23, increased susceptibility was observed for fungicides F2, F4, F5, and F6 following plasma expositions (Table 3 and Table 4). The MIC and MFC values for F2 in mycelia treated 15 times (P15) were reduced twofold compared to controls. For F4, a significant decrease in MFC was observed only after 15 treatments, from 5.0 mg mL^−1^ in P0 to 2.5 mg mL^−1^ in P15. No change was detected in MIC values for F5, which remained at 2.5 mg mL^−1^; however, MFC values decreased from 20 mg mL^−1^ in P0 and P5 to 10 mg mL^−1^ in P10 and further to 1.25 mg mL^−1^ in P15. The highest sensitivity to F6 was observed after five plasma treatments (P5), with MIC and MFC values of 1.95 × 10^−2^ mg mL^−1^. For P10 and P15, MIC values did not differ significantly from the control (3.91 × 10^−2^ mg mL^−1^), while the MFC values were two times lower.

#### 2.5.2. Effect of DBD Plasma on the Ability of the Tested Fungi to Form Biofilms

**Figure 5:**
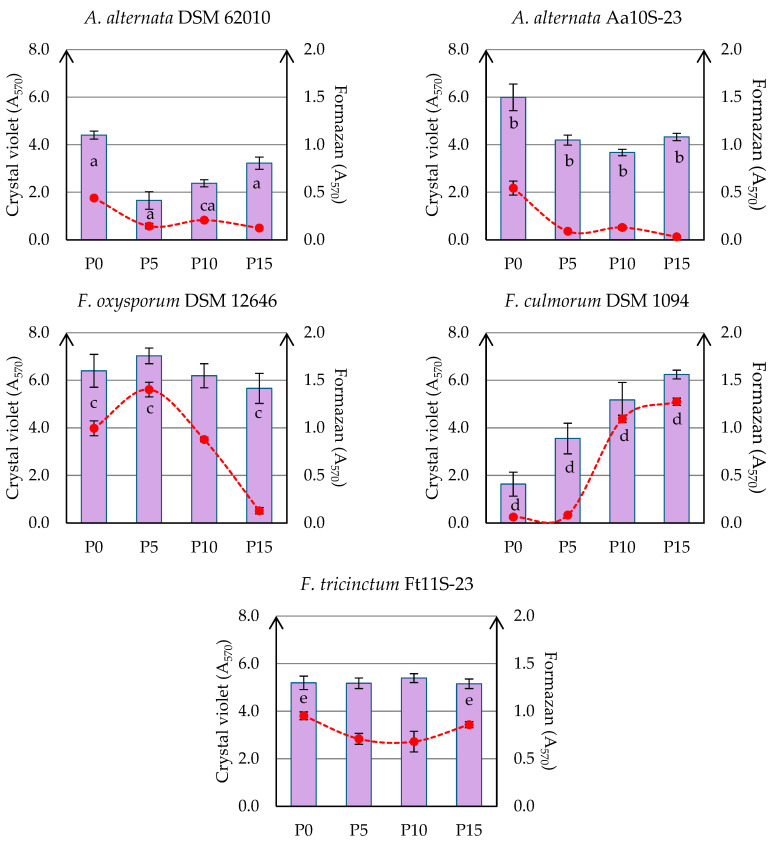
Biofilm formation capacities of the studied fungi subjected to multiple exposures to a sublethal dose of DBD plasma during a 24 h incubation period. The violet bars represent the biomass of the biofilms (left y-axis) based on the absorbance readings of crystal violet (A_570_), while the red points (right y-axis) indicate the cell mitochondrial metabolic activity determined using the MTT assay. a, b, c, d, and e indicate significant differences according to Dunnett’s post hoc test following one-way ANOVA (*p* < 0.05).

To assess the ability of the spores of the studied fungi to form biofilms, crystal violet staining was employed. The mitochondrial metabolic activity of the cells forming the biofilm structure was determined using the MTT assay. The obtained results are presented in Figure 5.

As shown in this figure, the strain *Fusarium oxysporum* DSM 12646 exhibited the highest capacity for biofilm formation prior to exposure to DBD plasma, as indicated by a high absorbance value of crystal violet (A_570_ = 6.40 ± 0.69). The metabolic activity of the mitochondria in the cells of this microorganism, as evidenced by a formazan absorbance of A_570_ = 0.996 ± 0.078, suggests a great number of viable cells present within the biofilm structure. Five plasma treatments of *F. oxysporum* DSM 12646 resulted in an increase in the absorbance value of crystal violet (A_570_ = 7.03 ± 0.33) and formazan (A_570_ = 1.40 ± 0.08) compared to the control (P0), after which these values gradually decreased following ten (P10) and fifteen (P15) plasma treatments (to 6.19 ± 0.051 and 0.878 ± 0.024 for P10, and 5.66 ± 0.63 and 0.129 ± 0.033 for P15, respectively).

*F. culmorum* DSM 1094 demonstrated the lowest biofilm formation capacity among the studied strains; however, this capacity significantly increased with the number of exposures to DBD plasma. The absorbance values of crystal violet gradually increased compared to the control (A_570_ = 1.64 ± 0.50) by 117% (P5), 217% (P10), and 282% (P15). Similarly, the mitochondrial metabolic activity of the cells forming the biofilm structure significantly increased relative to the control, initially by approximately 33% after five (P5) plasma treatments, and then by over 1600% after ten (P10) and over 1900% after fifteen (P15) plasma treatments.

A decrease in biofilm formation capacity due to multiple exposures to DBD plasma was observed for *A. alternata* DSM 62010 and *A. alternata* Aa10S–23. For *A. alternata* DSM 62010, the spores produced by the mycelium designated as P5 (after five exposures to plasma) exhibited the lowest biofilm formation capacity. After ten (P10) and fifteen (P15) plasma treatments, these values increased relative to P5 but remained lower compared to the control (P0) by approximately 46% and 53% for P10, and 27% and 72% for P15, respectively.

No changes were observed in the biofilm formation capacity of *F. tricinctum* Ft11S–23 following multiple exposures to DBD plasma.

## 3. Discussion

This study evaluated DBD plasma-induced alterations in morphological and physiological properties of molds from the genera *Alternaria* and *Fusarium*, which are significant pathogens affecting human and animal health. The observed changes in structural and functional characteristics of the tested microorganisms were induced by multiple exposures to sublethal doses of DBD plasma. In the experimental setup, live fungal cells (spores and mycelium) were deposited onto wooden disks to simulate growth on packing materials. Wood is generally regarded as suitable for use in the food industry from a hygienic perspective. It is important to note that the presence of mold on wooden packaging materials warrants monitoring, as fungal growth—including spore germination and hyphal development—can result in visible mycelium formation, rendering the wood esthetically unappealing and hygienically unsuitable for direct contact with food products. Contact with contaminated wooden packaging can serve as a transmission route for these pathogens to humans and animals.

Non-thermal atmospheric plasma is considered a promising alternative to traditional biocidal techniques, potentially overcoming many limitations associated with conventional disinfection methods [22,23,24,25]. This approach offers several environmental and sustainability advantages, including high efficacy at low temperatures, generation of active antimicrobial agents only during processing, absence of water or solvent consumption, no residual harmful by-products or wastewater, and the use of air as the working gas. The biocidal efficacy of non-thermal plasma depends on various factors, including device design and operational parameters such as gas composition, flow rate, ambient humidity and temperature, voltage, frequency, and the distance between the plasma source and the target surface [22]. The reactor used in our studies generates UV photons, electrons, ions and reactive oxygen/nitrogen species, which are highly effective as biocidal agents [20]. Additionally, the characteristics of the material being treated—such as surface properties, product type, and the type, concentration, and physiological state of microorganisms—also influence outcomes [22,23].

The fifteen-fold exposure of fungi to DBD plasma described in this study was conducted using a sublethal dose of plasma, as defined by the decimal reduction time (*δ*). This parameter is defined as the time required to achieve a tenfold reduction in the initial microbial population under specified conditions, corresponding to a 1-log decrease in microbial viability, i.e., the inactivation of 90% of the microorganisms [24]. It was shown that the decimal reduction time depended on the microorganism tested, and its value was found to range from 2 min 39 s for *F. culmorum* DSM 1094 to 5 min 19 s for *A. alternata* DSM 62010. These findings are consistent with previous studies indicating the efficacy of cold plasma in inactivating spores of various fungal genera, including *Aspergillus*, *Alternaria*, *Botrytis*, *Cladosporium*, *Fusarium*, and *Penicillium* [25,26,27,28,29].

Despite the encouraging findings related to the inactivation of filamentous fungi, a significant gap persists in the literature concerning the effects of non-thermal plasma on phenotypic and metabolic alterations, as well as on fungal pathogenicity. It has been reported that environmental stress can enhance the tolerance or resistance of fungi to specific stress factors [30]. Adaptive strategies in response to stress often induce morphological and physiological changes that enable these microorganisms to maintain viability under adverse conditions [31]. Understanding these mechanisms is essential for the development of effective non-thermal plasma treatment protocols.

Radial growth measurements of mycelium subjected to multiple plasma treatments demonstrated inhibition of vegetative growth in *F. tricinctum* Ft11S–23, no significant effect on the growth rate of colonies formed by *F. oxysporum* DSM 12646 and *A. alternata* DSM 62010, and a stimulatory effect on growth in *F. culmorum* DSM 1094. The absence of significant differences in colony diameter or the observed growth stimulation following multiple DBD plasma exposures may be attributed to cellular repair processes activated in response to plasma-induced damage. Due to the limited literature data on the effects of repeated plasma treatments of filamentous fungi, a direct comparison with other studies is impossible. However, some research examining fungal growth on solid media immediately after plasma treatment may provide insights. For instance, Julák et al. [32] investigated the growth of *A. oryzae* and *A. alternata* on solid media following conidial exposure to plasma. These authors proposed that the delayed colony growth is related to the plasma’s mechanism of action, primarily involving cell-wall damage. Cells exposed to sublethal plasma doses activate repair mechanisms that restore damaged cellular components and functions, thereby delaying growth and proliferation relative to untreated controls. These repair processes may account for the lack of significant differences in growth rates among *F. oxysporum* DSM 12646, *A. alternata* DSM 62010, and *A. alternata* Aa10S–23 after multiple plasma exposures compared to untreated fungi.

Conversely, the observed stimulatory effect of plasma on the growth rate of *F. culmorum* DSM 1094 is unexpected and has not been previously addressed in the scientific literature concerning filamentous fungi. However, existing studies indicate that the effects of DBD plasma on cells are contingent upon the levels of reactive oxygen species (ROS) produced during treatment, which are influenced by the treatment conditions and duration. Kalghatgi et al. [33] reported that low concentrations of ROS promote cell proliferation, whereas high ROS levels—produced by high-intensity or prolonged plasma treatments—can inhibit proliferation through mechanisms such as cell cycle arrest or apoptosis. It is important to note that these studies were conducted on human cells; therefore, the regulatory mechanisms governing fungal cell responses may differ.

Our experiments also demonstrated alterations in the tolerance of the tested microorganisms to oxidative stress. The minimum inhibitory concentration (MIC) and minimum fungicidal concentration (MFC) values for hydrogen peroxide against the examined fungi varied depending on the strain and the number of plasma exposures. A reduction in these parameters was observed following multiple treatments of the fungi with a sublethal dose of DBD plasma in *F. oxysporum* DSM 12646, *F. culmorum* DSM 1094, *A. alternata* DSM 62010, and *A. alternata* Aa10S–23, indicating decreased tolerance to oxidative stress. It was noted that this reduced tolerance was associated with the fungi’s growth rate on PDA medium. These findings are consistent with those reported by Zhong et al. [34], who demonstrated that strains of *Zymoseptoria tritici* exhibiting the fastest growth under controlled conditions (PDA) were the most sensitive to oxidative stress induced by hydrogen peroxide. Furthermore, these authors found no conclusive evidence linking melanization with increased tolerance of *Z. tritici* to oxidative stress.

However, an unexpected observation of increased resistance to hydrogen peroxide in *Fusarium tricinctum* Ft11S–23, which correlated with the increasing number of plasma exposures, was noted. The MIC values doubled after fifteen plasma treatments (P15). Meanwhile, the MFC increased progressively with increasing plasma exposure, rising from 25.5 mM for P0 to 102.0 mM for P15. Additionally, inhibition of mycelial growth on agar was observed as a consequence of repeated plasma treatment. At this stage of the research, this phenomenon is difficult to explain, as it has not yet been documented in the literature. Any speculation on this topic is premature, as it is essential to confirm these morphological and metabolic changes in other strains of this species. Our research team is actively working to expand the collection of these fungi.

Fungi are known to exhibit remarkable adaptability to fluctuating environmental conditions, including nutrient and water scarcity, as well as interspecies competition. These challenging conditions frequently induce oxidative stress, a phenomenon to which catalase, a ubiquitous enzyme in diverse fungal taxa, is a critical response. It is noteworthy that many fungal species possess multiple catalase isoforms, in addition to other hydrogen peroxide-scavenging enzymes [35]. The changes in the tolerance of the studied fungi resulting from repeated exposure to DBD plasma were also analyzed in relation to the levels of intracellular catalases. This study was conducted in an unconventional manner, as the cells were pre-incubated with hydrogen peroxide prior to the analysis of catalase activity levels. Consequently, the experiment did not reveal changes in the activity of constitutive catalases under the influence of plasma; rather, it should be interpreted as an effect of the expression of these proteins induced by the presence of the stressor. The results obtained showed that DBD plasma treatment modulated intracellular catalase level, though the resultant findings were not definitively conclusive. Specifically, it was noted an increase in catalase activity in *A. alternata* Aa10S–23 and *F. culmorum* DSM 1094 following repeated plasma exposure. However, no direct correlation was established between the frequency of DBD plasma exposure and the measured “total” catalase activity within the fungal cells. A significant limitation of this study lies in the methodology employed for determining catalase activity. Consequently, future investigations focusing on the analysis of individual catalase isoforms present in fungal cells are warranted to provide a more precise understanding of DBD plasma’s effects on this crucial enzymatic system.

It is well known that fungicides exert their effects by specifically disrupting critical cellular processes within pathogenic fungi, such as maintaining plasma membrane integrity, a cell division, or mitochondrial respiration [36]. Fungicide resistance can arise from mutations in genes encoding fungicide targets, a phenomenon known as qualitative resistance. Resistance can be inherited and become a stable trait within fungal populations. Resistant mutants may become dominant, leading to decreased fungicide efficacy and complicating disease management efforts [37]. Considering the phenomenon of qualitative resistance, it seems reasonable to examine the effect of DBD plasma on the tolerance of the tested fungi to known fungicides. Six commercially available fungicides were tested in our experiments. Three of these (trade names: Magnicur Energy, SCORPION 325, and SWITCH 62.5) were two-component formulations containing, respectively, fosetyl-Al. (a phosphonate) combined with propamocarb hydrochloride (a carbamate), azoxystrobin (a strobilurin) combined with difenoconazole (a triazole), and cyprodinil (an anilinopyrimidine) combined with fludioxonil (a phenylpyrrole). The remaining three fungicides contained: tebuconazole (a triazole), trihydroxydicopper chloride, or dodine (1-dodecylguanidine acetate). The results obtained demonstrated that repeated exposure to DBD plasma resulted in a decreased tolerance of the tested fungi to at least one fungicide. Concurrently, all strains exhibited a reduction in tolerance to trihydroxydicopper chloride (Miedzian 50 WP). An increase in tolerance was observed in only four cases: *F. culmorum* DSM 1094 against tebuconazole (Tobias-Pro EW) and azoxystrobin plus difenoconazole (Scorpion 325 S.C.), and *F. oxysporum* DSM 12646, and *A. alternata* DSM 62010 against dodine (Syllit 65 WP). The phenomenon of fungal sensitization to commercial biocides induced by repeated exposure to a sublethal doses of plasma is worth noting as a new potential application of DBD plasma for controlling resistant strains.

This study also evaluated the impact of multiple treatments with a sublethal dose of DBD plasma on the biofilm formation capacity of the studied fungi. While filamentous fungi are infrequently classified as biofilm-forming organisms—likely due to their failure to meet the conventional bacterial biofilm definition—they exhibit biofilm-like characteristics as outlined by Harding et al. [38]. These characteristics include the production of extracellular polymeric substances (EPS), intercellular communication through extracellular signals, differential gene expression, and enhanced resistance to antifungal agents compared to dispersed or loosely associated (planktonic) cells. The capacity for biofilm formation confers a survival advantage to fungal pathogens under unfavorable conditions and enhances resistance to antimicrobials [39].

The spore adhesion and biofilm formation capabilities of the tested fungi were assessed by measuring biofilm biomass (crystal violet staining). The results obtained indicated that *F. oxysporum* DSM 12646 spores exhibited the highest biofilm formation capacity; however, this ability diminished following ten and fifteen exposures to plasma. Similarly, *A. alternata* DSM 62010 and *A. alternata* Aa10S–23 demonstrated a reduced capacity for biofilm formation after multiple DBD plasma exposures.

In contrast, *F. culmorum* DSM 1094 spores initially displayed low biofilm formation ability, which significantly increased following multiple exposures to plasma. Analysis of changes in the metabolic activity of mitochondrial enzymes, measured using the MTT assay, revealed an unusually high increase in the concentration of formazan formed in the fungal cells after 10 and 15 plasma treatments. This phenomenon should not be attributed solely to the number of viable cells in the biofilm structure; rather, it may indicate damage to mitochondrial metabolism. At this stage of the research, providing an explanation for this observation would be speculative, as it cannot be adequately addressed without additional analyses (metabolomic and genotypic). Therefore, this issue remains an open question at this stage of the investigation.

Generally, our findings demonstrated that DBD plasma influences the biofilm formation capacity of fungal cells, with the observed changes depending on the pathogen species and the number of plasma treatments. Based on the conducted experiments, it can be inferred that multiple treatments of fungi with DBD plasma may either decrease or increase the spores’ capacity for biofilm formation. This phenomenon should be evaluated on an individual basis, taking into account not only the species of the microorganism, but also the specific strain. The ability of cells exposed to cold plasma to form biofilms was also investigated by Ebrahimi-Shaghaghi et al. [40]. These authors reported an inhibitory effect of cold plasma generated from a mixture of 98% helium and 2% oxygen on biofilm formation by *Candida albicans* ATCC 10231 in a time-dependent manner. Cells treated with plasma for 210 s exhibited a significant reduction (65.9%) in biofilm formation capacity compared to the untreated control. However, this study did not consider the reduction in the number of viable cells resulting from plasma exposure, which may have significantly influenced the observed decrease in biofilm formation ability.

It is generally believed that sublethal doses of biocides can induce biofilm production by microorganisms [39]. It is postulated that antimicrobial agents may lead to the selection of more resistant subpopulations capable of forming more robust biofilms, which complicates the elimination of these pathogens. The ability of bacterial populations to adapt to changing environmental conditions is fundamentally dependent on their phenotypic diversity.

Concluding our discussion regarding the effects of DBD plasma on fungal morphology/physiology, antifungal sensitivity, and biofilm formation, there is a noteworthy phenomenon called hormesis, which describes a biphasic response of fungal cells to sublethal levels of oxidative stress that induce adaptive physiological changes. The phenomenon of hormesis has been summarized in excellent reviews [41,42,43]. These adaptive mechanisms can be diverse, and the role of metabolic pathways in the fungal response to oxidative stress is not uniformly conserved, indicating that there are always species aspects to the elicited response.

It was shown that fungal cells exposed to mild oxidative stress (e.g., low concentrations of hydrogen peroxide (H_2_O_2_) or sublethal doses of fungicides) respond by increasing their overall vitality, and this pretreatment with stressors can completely prevent cell death resulting from subsequent exposure to lethal levels of oxidative biocides. Thus, low doses of oxidative stress can function as chemical signals, stimulating fungal cells to activate highly conserved signaling pathways, such as the de novo synthesis of key protective enzymes, including catalase, superoxide dismutase, and glutathione peroxidase. Oxidative stress not only alters internal chemistry but also visibly affects the growth and structure of mycelia. Studies have shown that fungi exposed to oxidizing agents can develop shorter, more branched hyphae, resulting in a reduction in the overall size of their growth units. The cellular response to oxidative stress is primarily mediated by conserved bZIP (basic leucine zipper) transcription factors, particularly the regulator Yap1, which controls the transport and expression of antioxidant genes. Furthermore, the HOG (high osmolarity glycerol) pathway coordinates responses throughout the fungal kingdom.

The observed challenges associated with dielectric barrier discharge plasma activity represent a significant consideration in the industrial application of this technique for the eradication of pathogenic fungi. Our studies indicate that multiple exposures of fungi to non-thermal plasma does not invariably lead to adaptation to environmental conditions, nor does it result in the selection of more resistant subpopulations. This phenomenon underscores the uniqueness of DBD plasma in the destruction of mold fungi without promoting the emergence of population resistance. However, the potential for enhanced tolerance to fungicides and/or oxidizing stress induced by cold plasma should not be overlooked. This phenomenon may result in increased virulence of phytopathogenic fungi, which could subsequently affect food losses and food security for both humans and animals. Ongoing research in this domain is being conducted by our research group.

## 4. Materials and Methods

### 4.1. Fungal Strains and Culture Conditions

The tested fungal strains were obtained from the German Collection of Microorganisms and Cell Cultures at the Leibniz Institute (DSMZ) (Braunschweig, Germany) and stored freeze-dried at −70 °C (*Fusarium culmorum* DSM 1094, *Alternaria alternata* DSM 62010) or as active cultures (*Fusarium oxysporum* DSM 12646) at 4 °C.

### 4.2. Isolation and Characterization of Wild Strains

Wild filamentous fungi were isolated from the surfaces of wooden crates used for storing vegetables (specifically potatoes) on farms near Środa Wielkopolska, Poland. Samples were taken from areas exhibiting visible mold growth on the vegetable storage crates using contact plates containing Sabouraud Glucose + Chloramphenicol medium (GRASO Biotech, Owidz, Poland). The plates were incubated at 25 °C for 7 to 10 days. Following incubation, pure colonies were isolated through single spore isolation or by excising the hyphal tip from the colony margin, as described by Chang et al. [44]. The fungal isolates were subsequently sub-cultured onto potato dextrose agar (PDA) (BTL LLC, Łódź, Poland) and incubated at 25 °C for an additional 7 days. Initial classification of the isolates was conducted based on morphological characteristics, including pigmentation, shape, elevation, and colony margins, as well as spore and conidiophore features, in accordance with a standard taxonomic system [45].

Species identification of the selected isolates was performed using a molecular method based on polymerase chain reaction (PCR) with PCR Mix Plus reagents (A&A Biotechnology, Gdańsk, Poland). For the wild strains, mycelia were collected from a ten-day culture on PDA medium (rinsed with sterile water, lyophilized, and homogenized). DNA isolation was performed using the method outlined by Doyle and Doyle [46], with modifications. The DNA was suspended in water and diluted to a concentration of 10 ngµL^−1^. Preliminary identification of *Alternaria* spp. and *Fusarium* spp. isolates was based on the sequencing of the ITS regions using primers ITS1 and ITS4 [47]. Further species-specific analyses were conducted using primers ITS5 and MR for *Alternaria* sp. [48], and primers tri1 and tri2 for *Fusarium* sp. [49] according to the reaction conditions described by their authors. The expected product sizes were 505 bpand 215 bp, respectively. The ITS sequences of the isolates have been deposited in the NCBI GenBank database.

Cultures of strains isolated from the surfaces of wooden boxes were established by inoculating 100 mL of liquid medium with mycelium collected from the edge of a pure culture on potato dextrose agar (PDA) using a sterile loop. The inoculated medium was subsequently incubated on a shaker at 120 rpm and 25 °C for 7 days. Following this incubation period, 100 µL of the initial culture was removed and plated onto PDA medium. The plates were incubated at 25 °C for an additional 7 days and were then stored at 4 °C for a maximum of 28 days.

### 4.3. Plasma Reactor with Dielectric Barrier Discharge

Plasma treatment of the studied fungi was carried out using a parallel-plate DBD reactor designed by Dr Eng. Tomasz Czapka (Faculty of Electrical Engineering, Department of Electrical Engineering and Electrical Technology, Wrocław University of Science and Technology, Poland). A dielectric barrier plasma reactor featuring a plane-parallel geometry and an asymmetric electrode configuration was employed in this study. The discharge system comprised two aluminum electrodes separated by a single dielectric layer, which consisted of a 1 mm thick Al_2_O_3_ plate. This dielectric layer was positioned on a grounded electrode. Each electrode had an approximate surface area of 120 cm^2^. The air gap, where electrical discharges occurred as pulsed and intermittent discharges, was maintained at a width of 4 mm. The reactor operated at atmospheric pressure, utilizing atmospheric air as the working gas.

The reactor was powered by a high-voltage frequency-modulated pulse generator (Dora PS, Wrocław, Poland). The pulse discharge parameters included an amplitude of 5.5 kV, a frequency of 38 kHz, and a power output of 12 W. The average power density delivered to the samples was 75 mW cm^−2^. The characteristics of the plasma generated by this reactor have been detailed in our previous publications [21,50]. Test samples, specifically wooden disks, were placed on the surface of the dielectric layer during plasma exposure. A diagram of the plasma reactor utilized in this study is presented in Figure S4.

### 4.4. Fungal Cell Inactivation by DBD Plasma

A mycelial suspension was prepared according to the procedure described previously [21,50], with slight modifications. Specifically, 10 mL of sterile deionized water supplemented with 0.1% Tween 80 was applied on the surface of seven-day-old pure cultures of the studied phytopathogenic fungi grown on PDA medium. The mycelium and spores were gently scraped off using a sterile loop and the resulting suspension was thoroughly mixed on a laboratory shaker for 7 min. After thorough mixing, all suspensions were filtered through a double layer of sterile gauze to remove larger mycelial fragments. The cell suspension was then centrifuged at 7500 rpm (6918× *g*) for 7 min and diluted with sterile deionized water to achieve a final concentration of 10^6^ CFU mL^−1^, as determined using a Howard counting chamber. A hemocytometer was also used to calculate the ratio of mycelial cells to spores. For each working inoculum, the ratio of mycelial cells to spores was individually determined and it was shown that this ratio was approximately 9:1 for *A. alternata* DSM 62010 and *A. alternata* Aa10S–23, and 1:1 for *F. oxysporum* DSM 12646, *F. culmorum* DSM 1094, and *F. tricinctum* Ft11S–23, respectively. The concentration of each inoculum was confirmed through serial tenfold dilutions and subsequent plating onto PDA medium.

Metabolically active mycelium was employed to evaluate the response complexity of fungal cells to DBD plasma treatment. Sterile wooden disks (diameter 8 mm; thickness 1 mm) were placed individually in Petri dishes and artificially contaminated by applying 20 µL (10^6^ CFU mL^−1^) of the fungal suspension onto their surfaces. The disks were allowed to dry for 24 h at room temperature. Then, wooden disks, artificially inoculated with the fungal suspension, were placed individually in the central part of the DBD reactor (this reactor designed by Dr Eng. Tomasz Czapka (Faculty of Electrical Engineering, Department of Electrical Engineering and Electrical Technology, Wrocław University of Science and Technology, Poland)) between the electrodes and exposed to plasma (only the contaminated side of each sample was in direct contact with the plasma). Disks contaminated with the fungal suspension that were not exposed to plasma served as controls (t = 0).

Plasma-treated (and non-treated) disks were transferred to tubes containing 1 mL of sterile deionized water and mixed thoroughly using a laboratory shaker. Viable cell counts were determined using a tenfold serial dilution method followed by plating on PDA medium. Plates were incubated in the dark at 25 °C for 24 to 72 h, until mycelial growth was observed. Results were expressed as colony-forming units (CFUs) per cm^2^ of disk surface. Each experiment was conducted in triplicate.

### 4.5. Procedure for Multiple Treatments of the Studied Fungal Cells with a Sublethal Dose of DBD Plasma

The procedure for the repeated exposure of filamentous fungi to DBD plasma has been detailed in reference [50]. However, to enhance the reader’s understanding of the results obtained, it is presented below with minor modifications.

**Figure 6:**
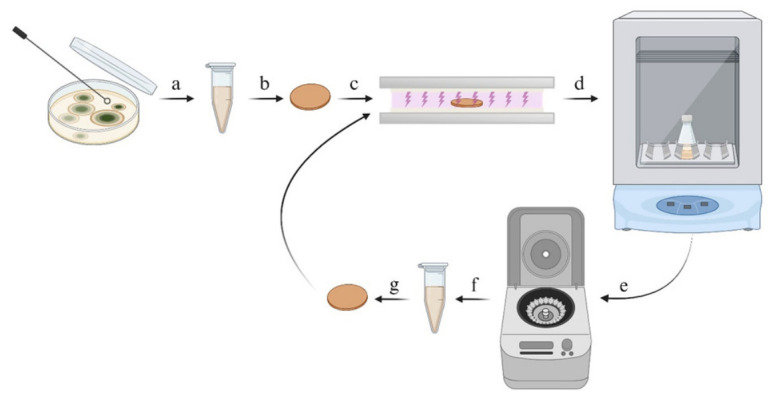
Schematic diagram of the procedure for multiple treatments of the studied fungal cells with a sublethal dose of DBD plasma. A description of the individual steps is provided in the text. Prepared based on [50].

The procedure for the multiple treatments of the studied fungi with DBD plasma was conducted using a sublethal dose corresponding to the δ-value for fungi inactivated on wood surfaces (see Table 1). The experimental procedure is illustrated in Figure 6.

A fungal suspension with a cell concentration of 10^6^ CFU mL^−1^ was prepared (a), and the surface of a wooden disk was artificially contaminated according to the procedure described in Section 4.4 (b). The contaminated disk was then exposed to a sublethal dose of DBD plasma (c). Subsequently, the disk was transferred to a 250 mL Erlenmeyer flask containing 50 mL of PDA medium and incubated on a laboratory shaker at 25 °C and 120 rpm (d). After 48 h of incubation, 2 mL of the culture was removed and centrifuged at 7500 rpm (6918× *g*) for 5 min (e). The supernatant was discarded, and the remaining mycelium was suspended in sterile deionized water to achieve a final cell concentration of 10^6^ CFU mL^−1^ using a Howard counting chamber (f). This prepared suspension was then used to inoculate another disk, which was allowed to dry at room temperature for 24 h (g). The contaminated disk was subsequently exposed to another sublethal dose of plasma (h). This procedure was repeated for a total of 15 cycles. Mycelium not treated with DBD plasma was prepared in the same manner, omitting the step marked as (c).

Fungal cells were then suspended in sterile deionized water to achieve a final concentration of 10^6^ CFU mL^−1^. The prepared suspensions were stored at 4 °C for subsequent studies for no longer than 7 days.

### 4.6. Assessment of the Growth Rate of Fungi Subjected to Multiple Exposures to DBD

A 10 µL aliquot of a fungal suspension (10^6^ CFU mL^−1^) obtained without DBD plasma treatment (P0) and after the fifth (P5), tenth (P10), and fifteenth (P15) treatments, prepared according to the procedure described in Section 4.4 and Section 4.5, was applied to the center of a plate containing solid PDA medium. The plates were incubated at 25 °C in the dark. Fungal colony diameters were measured according to the procedure described by Wang et al. [29] after 3, 5, 7, and 14 days of incubation. The experiment was conducted twice, with two replicates for each sample.

### 4.7. Investigating the Effect of Multiple Exposures of Fungi to DBD Plasma on Cellular Tolerance to Oxidative Stress

#### 4.7.1. Determination of MIC/MFC Values for Hydrogen Peroxide

Minimum inhibitory concentrations (MICs) of hydrogen peroxide were determined by the microdilution method in culture medium as described by Cobrado et al. [51], with minor modifications. Fungal suspensions without plasma treatment (P0) and after multiple plasma expositions (P5, P10, P15) were prepared according to the methodology described in Section 4.4 and Section 4.5. The suspensions were then centrifuged and resuspended in an equivalent volume of fresh Potato Dextrose Broth (PDB). A stock solution of hydrogen peroxide was prepared in double-concentrated PDB medium at a concentration of 6540 mM (the concentration of the prepared working solution was calibrated spectrophotometrically using ammonium monovanadate). Next, 100 µL of fungal suspension and 100 µL of hydrogen peroxide were added to each well of a 96-well plate (F.L. Medical, Italy) to obtain final concentrations of H_2_O_2_ ranging from 3270 mM to 3.19 mM. Plates were incubated at 25 °C for 48–72 h. A control group consisted of a fungal suspension with PDB (without hydrogen peroxide). The MIC was defined as the lowest concentration of H_2_O_2_, whichresulted in no visible fungal growth in the well. This study was conducted in two independent experiments, with each experiment repeated three times (*n* = 6).

Minimum fungicidal concentrations (MFCs) of hydrogen peroxide were determined for untreated fungi (P0) and fungi treated with DBD plasma (P5, P10, P15). After 24 h of incubation, 10 µL of fungal suspension was removed from each well of the 96-well plate and plated onto a PDA medium. The plates were then incubated at 25 °C until mycelial growth was observed in the control group (usually 24–48 h). The MFC was defined as the lowest concentration of hydrogen peroxide that completely inhibited fungal growth on the solid medium. This study was conducted in two independent experiments, with each experiment repeated three times (*n* = 6).

#### 4.7.2. Determination of Catalase Activity

Crude fungal extracts for catalase activity assays were prepared from P0, P5, P10, and P15 cultures adapted to 1.59 mM H_2_O_2_. For adaptation, cells were incubated with H_2_O_2_ for 60 min at room temperature (20 °C). Following incubation, the cells were washed with sterile distilled water and pelleted by centrifugation (8500 rpm/~8880× *g*; 5 min; 4 °C). The pellet was resuspended in 50 mM phosphate buffer (pH 7.0) containing 0.5 mM phenylmethylsulfonyl fluoride (PMSF). The cell suspension was sonicated on ice (four 10 s pulses), and the resulting homogenate was centrifuged at 17,000 rpm (~35,500× *g*) for 45 min at 4 °C. The supernatant (cell-free extract) was collected used to determine catalase activity.

The activity of catalase was determined using the method described by Montejo et al. [52]. The concentration of H_2_O_2_ substrate solution was 10 mM; time of incubation was 10 min at room temperature (20 °C). This study was conducted in two independent experiments, with each experiment repeated three times (*n* = 6).

### 4.8. Studies on Development of Tolerance to Fungicides After Multiple Treated with DBD Plasma

Fungal suspensions were prepared in medium according to the methodology described in Section 4.4. The MICs of commercially available fungicides were determined using the microdilution method in a 96-well plate according to the method described in Section 4.7. The range of tested concentrations of fungicides was determined based on the manufacturer’s maximum doses listed on the label and recommendations for the specific plant disease. Fungicides (F) labeled F1–F3 were liquid concentrates of the active ingredients. Stock solutions of these agents were prepared by dilution in sterile deionized water. These fungicidal products contained the following active substances: fosetyl-aluminum (31%) and propamocarb hydrochloride (25.0%, 530 g/L) for F1; tebuconazole (27.0%) for F2; and azoxystrobin (18.2%) and difenoconazole (11.4%) for F3.

Fungicides labeled F4–F6 were powders. These fungicidal products comprised the following active ingredients: trihydroxy dicopper chloride (87.5%) for F4; cyprodynil (37.5%) for F5; and fludioksonil (25%) and dodyna (65%) for F6.

Stock solutions of all these agents were prepared by weighing the appropriate amount of powder and dissolving it in sterile deionized water or suspending it in sterile deionized water with 1% agar–agar to maintain a uniform concentration of active ingredients throughout the volume and prevent settling at the bottom of the well.

The active ingredient content (as provided by the manufacturer) and the tested concentration range of the antifungal agents are provided in Table 5.

**Table 5:** Tested range of concentrations for the fungicide product.

Fungicide Product/Manufacturer	Designation	Strains	
		*A. alternata* DSM 62010 *A. alternata* Aa10S-23	*F. oxysporum* DSM 12646 *F. culmorum* DSM 1094 *F. tricinctum* Ft11S-23
		Tested Product Concentration Range	
Magnicur Energy (Protect Garden)	F1	40–0.625 µL mL^−1^	40–0.625 mg mL^−1^
Tobias-Pro 250 EW (Procam)	F2	0.313–4.88 × 10^−3^ µL mL^−1^	0.313–4.88 × 10^−3^ mg mL^−1^
SCORPION 325 S.C. (Agrecol)	F3	0.313–4.88 × 10^−3^ µL mL^−1^	40–0.625 mg mL^−1^
MIEDZIAN 50 WP (Target)	F4	40–0.625 µL mL^−1^	40–0.625 mg mL^−1^
SWITCH 62.5 WG (Agrecol)	F5	0.313–4.88 × 10^−3^ µL mL^−1^	40–0.625 mg mL^−1^
SYLLIT 65 WP (Agrecol)	F6	0.313–4.88 × 10^−3^ µL mL^−1^	0.313–4.88 × 10^−3^ mg mL^−1^

This study was conducted in two independent experiments, with each experiment repeated three times (*n* = 6).

### 4.9. Effect of DBD Plasma on Adhesion and Biofilm Formation Ability of Fungal Spores

#### 4.9.1. Biofilm Formation

The biofilm-forming capacity of the spores produced by fungi exposed to multiple DBD plasma treatments was assessed using the procedure described by Siqueira and Lima [53]. Spores of each strain were collected by adding 10 mL of physiological saline supplemented with 0.1% Tween to the plate and scraping the spores from the surface of the medium using a sterile loop. The resulting fungal suspension was thoroughly mixed on a laboratory shaker and then filtered through a double layer of sterile gauze to remove mycelial fragments. The cell suspension was then centrifuged at 7500 rpm (6918× *g*) for 5 min and diluted with PDB medium to a concentration of 10^5^–10^6^ CFU mL^−1^ (spore counts were estimated using a Howard cell). The spore concentration in each suspension was confirmed by serial tenfold dilutions method and plating on PDA medium. This study was conducted in two independent experiment, with each experiment repeated three times (*n* = 6).

Two hundred and fifty microliters of fungal suspension and 250 μL of PDB medium were added to the wells of 24-well flat-bottom plates (Biologix, Hallbergmoos, Germany). The plates were incubated at 25 °C for 24 h to allow biofilm formation. The culture medium, including non-adherent cells, was then removed, and the resulting biofilms were gently washed with sterile saline and allowed to dry. The ability of the tested strains to form biofilms on polystyrene surfaces was determined by crystal violet staining, and the metabolic activity of the formed biofilms was assessed using the MTT assay, according to the procedure described by Siqueira and Lima [53] with minor modifications. A 0.5 mg mL^−1^ solution of (3-(4,5-dimethylthiazol-2-yl)-2,5-diphenyltetrazolium bromide) was prepared by dissolving the powder in sterile PBS buffer, pH 7.4. The reagent was stored at 4 °C in a dark glass bottle. A 500 µL MTT solution was added to the wells containing the formed and washed biofilm. The plates were then incubated in a laboratory incubator at 25 °C for 4 h. After this time, 500 µL of acidic isopropanol (0.04 M hydrochloric acid in propan-2-ol) was added to each well to dissolve the formed formazan crystals. The metabolic activity of biofilm-forming fungi was assessed by measuring formazan absorbance at a wavelength of λ = 570 nm using a Cary UV–Vis spectrophotometer (Agilent Technologies Inc., Santa Clara, CA, USA). This study was conducted in two independent experiment, with each experiment repeated three times (*n* = 6).

#### 4.9.2. Crystal Violet Staining

Five hundred microliters of a 0.05% (*w*/*v*) aqueous crystal violet solution was added to the wells containing the formed and washed biofilms and incubated for 30 min at 25 °C. After incubation, the dye was removed, and the biofilm was washed with sterile deionized water. Five hundred microliters of ethanol (96%) was added to each well, and the absorbance of the dissolved crystal violet was measured at a wavelength of λ = 570 nm using a Cary UV–Vis spectrophotometer (Agilent Technologies Inc., Santa Clara, CA, USA). This showed that the crystal violet absorbance value was proportional to the biofilm biomass, which consists of hyphae and extracellular polymeric material. This study was conducted in two independent experiments, with each experiment repeated three times (*n* = 6).

### 4.10. Statistical Analysis

In the statistical analysis, one-way analysis of variance (ANOVA) was used to assess differences between the study groups. For multiple comparisons (each time relative to the control group) Dunnet’s post hoc test was used, which allowed for the reduction in Type I error inflation while maintaining the control group as the reference point.

Before conducting the analysis, the assumptions of the ANOVA test were verified, including the normality of the data distribution and the homogeneity of variances, which were found to be met. A statistical significance level of α = 0.05 (95% confidence level) was adopted. Results were considered statistically significant for *p*-values < 0.05. The statistical analyses of the data obtained in the experiments were performed using the STATISTICA data analysis software package (Statistica version 13.3, StatSoft Inc., Kraków, Poland).

Statistical analysis of the determined MIC/MFC values was not performed because uniform results were obtained across many time groups studied, with all observations yielding identical values and resulting in zero within-group variance. Additionally, in some experiments, these parameters could not be determined as they fell outside the range of tested concentrations. The ordinal nature of the data further limited the applicability of statistical analysis. Therefore, the results are presented solely in descriptive form.

## Figures and Tables

**Table 1:** The decimal reduction time (δ) of the filamentous fungi exposed to DBD plasma.

Decimal Reduction Time	Strains				
	*F. oxysporum* DSM 12646	*F. culmorum* DSM 1094	*F. tricinctum* Ft11S–23	*A.alternata* DSM 62010	*A. alternata* Aa10S–23
*δ* *	3 min 28 s	2 min 39 s	3 min 11 s	5 min 19 s	4 min 26 s

* The *δ* values calculated for the strains *F. oxysporum* DSM 12646, *F. culmorum* DSM 1094 and *A*. *alternata* DSM 62010 were taken from [21].

**Table 2:** Minimum inhibitory concentration (MIC) and minimum fungicidal concentration (MFC) of hydrogen peroxide against studied fungi multiple treated with a sublethal dose of DBD plasma (P5, P10, P15). The control was the sensitivity to H_2_O_2_ of mycelium not treated with DBD plasma (P0).

Strain	Number of Exposures to DBD Plasma			
	P0	P5	P10	P15
	MIC/MBC (mM)			
*A. alternata* DSM 62010	25.5/102.0	25.5/102.0	12.8/51.0	12.8/51.0
*A. alternata* Aa10S–23	12.8/51.0	12.8/51.0	6.38/51.0	6.38/25.5
*F. oxysporum* DSM 12646	12.8/51.0	12.8/51.0	12.8/51.0	12.8/25.5
*F. culmorum* DSM 1094	51.0/100.0	12.8/51.0	12.8/51.0	12.8/51.0
*F. tricinctum* Ft11S–23	6.38/25.5	6.38/51.0	6.38/51.0	12.8/102.0

**Table 3:** The minimum inhibitory concentration (MIC) and minimum fungicidal concentration (MFC) values of selected fungicides F1–F3 against the tested fungi.

Strain	Number of Plasma Treatment	Magnicur Energy F1	Tobias-Pro 250 EW F2	SCORPION 325 S.C. F3
	MIC/MFC (µL mL^−1^)			
*A. alternata* DSM 62010	P0	40.0/>40	<4.88 × 10^−3^/0.156	<4.88 × 10^−3^/1.95 × 10^−2^
	P5	10.0/>40	<4.88 × 10^−3^/0.156	<4.88 × 10^−3^/1.95 × 10^−2^
	P10	5.0/>40	<4.88 × 10^−3^/0.156	<4.88 × 10^−3^/1.95 × 10^−2^
	P15	2.5/20.0	<4.88 × 10^−3^/7.81 × 10^−2^	<4.88 × 10^−3^/1.95 × 10^−2^
*A. alternata* Aa10S–23	P0	>40/>40	<4.88 × 10^−3^/0.313	<4.88 × 10^−3^/3.91 × 10^−2^
	P5	>40/>40	<4.88 × 10^−3^/0.313	<4.88 × 10^−3^/3.91 × 10^−2^
	P10	>40/>40	<4.88 × 10^−3^/0.313	<4.88 × 10^−3^/3.91 × 10^−2^
	P15	20.0/20.0	<4.88 × 10^−3^/0.313	<4.88 × 10^−3^/3.91 × 10^−2^
*F. oxysporum* DSM 12646	P0	40.0/>40	<4.88 × 10^−3^/0.156	1.00/20.0
	P5	10.0/>40	0.156/0.313	10.0/20.0
	P10	10.0/>40	0.156/0.313	10.0/20.0
	P15	10.0/>40	0.156/0.313	10.0/20.0
*F. culmorum* DSM 1094	P0	>40/>40	<4.88 × 10^−3^/0.156	<0.625/2.0
	P5	>40/>40	0.156/0.313	20.0/>40.0
	P10	>40/>40	0.156/0.313	20.0/>40.0
	P15	>40/>40	0.156/0.313	20.0/>40.0
*F. tricinctum* Ft11S–23	P0	>40/>40	0.156/0.313	2.5/10.0
	P5	>40/>40	7.81 × 10^−2^/0.313	2.5/10.0
	P10	>40/>40	7.81 × 10^−2^/0.156	2.5/10.0
	P15	>40/>40	7.81 × 10^−2^/0.156	2.5/10.0

**Table 4:** The minimum inhibitory concentration (MIC) and minimum fungicidal concentration (MFC) values of fungicides F4–F6 against the tested fungi.

Strain	Number of Plasma Treatment	MIEDZIAN 50 WP F4	SWITCH 62.5 WG F5	SYLLIT 65 WP F6
	MIC/MFC (mg mL^−1^)			
*A. alternata* DSM 62010	P0	2.5/>40	<4.88 × 10^−3^/3.91 × 10^−2^	<4.88 × 10^−3^/<4.88 × 10^−3^
	P5	2.5/>40	<4.88 × 10^−3^/1.95 × 10^−2^	<4.88 × 10^−3^/9.77 × 10^−3^
	P10	2.5/5.0	<4.88 × 10^−3^/1.95 × 10^−2^	<4.88 × 10^−3^/9.77 × 10^−3^
	P15	2.5/2.5	<4.88 × 10^−3^/1.95 × 10^−2^	<4.88 × 10^−3^/9.77 × 10^−3^
*A. alternata* Aa10S–23	P0	2.5/>40	<4.88 × 10^−3^/9.77 × 10^−3^	<4.88 × 10^−3^/1.95 × 10^−2^
	P5	2.5/40	<4.88 × 10^−3^/9.77 × 10^−3^	<4.88 × 10^−3^/9.77 × 10^−3^
	P10	2.5/40	<4.88 × 10^−3^/9.77 × 10^−3^	<4.88 × 10^−3^/9.77 × 10^−3^
	P15	1.25/5.0	<4.88 × 10^−3^/9.77 × 10^−3^	<4.88 × 10^−3^/<4.88 × 10^−3^
*F. oxysporum* DSM 12646	P0	2.5/>40	40.0/40.0	3.91 × 10^−2^/3.91 × 10^−2^
	P5	2.5/10.0	40.0/40.0	3.91 × 10^−2^/7.81 × 10^−2^
	P10	2.5/10.0	40.0/40.0	7.81 × 10^−2^/0.156
	P15	2.5/10.0	40.0/40.0	7.81 × 10^−2^/0.313
*F. culmorum* DSM 1094	P0	<0.625/40.0	40.0/>40	0.313/0.625
	P5	<0.625/10.0	20.0/40.0	1.95 × 10^−2^/3.91 × 10^−2^
	P10	<0.625/10.0	<0.625/5.0	3.91 × 10^−2^/7.81 × 10^−2^
	P15	<0.625/10.0	<0.625/2.5	3.91 × 10^−2^/7.81 × 10^−2^
*F. tricinctum* Ft11S–23	P0	<0.625/5.0	2.5/20.0	3.91 × 10^−2^/7.81 × 10^−2^
	P5	<0.625/5.0	2.5/10.0	1.95 × 10^−2^/1.95 × 10^−2^
	P10	<0.625/5.0	2.5/10.0	1.95 × 10^−2^/3.91 × 10^−2^
	P15	<0.625/2.5	2.5/5.0	1.95 × 10^−2^/3.91 × 10^−2^

## Data Availability

Data will be available upon reasonable request.

## Supplementary Materials

The following supporting information can be downloaded at: https://www.mdpi.com/article/10.3390/molecules31142422/s1. Figure S1: The morphology of *F. oxysporum* DSM 12646 colonies was assessed in untreated samples (P0) and after five (P5), ten (P10), and fifteen (P15) DBD plasma treatments on potato dextrose agar (PDA) over a 14-day culture period. The upper portion of each image displays the colony’s obverse view, while the lower portion presents the reverse view. Images were captured after 3, 5, 7, and 14 days of incubation. Figure S2: The morphology of *F. culmorum* DSM 1094 colonies was assessed in untreated samples (P0) and after five (P5), ten (P10), and fifteen (P15) DBD plasma treatments on potato dextrose agar (PDA) over a 14-day culture period. The upper portion of each image displays the colony’s obverse view, while the lower portion presents the reverse view. Images were captured after 3, 5, 7, and 14 days of incubation. Figure S3: The morphology of *F. tricinctum* Ft11S-23 colonies was assessed in untreated samples (P0) and after five (P5), ten (P10), and fifteen (P15) DBD plasma treatments on potato dextrose agar (PDA) over a 14-day culture period. The upper portion of each image displays the colony’s obverse view, while the lower portion presents the reverse view. Images were captured after 3, 5, 7, and 14 days of incubation. Figure S4. Schematic view of a plasma system using dielectric barrier discharges (DBD) in the air as the working gas, which was applied for multiple treatments of the studied plant pathogens. The plasma reactor was powered by a high-voltage pulse generator with a modulated frequency (Dora PS, Wroclaw, Poland). The pulse amplitude, frequency, and discharge power were 5.5 kV, 38 kHz, and 9 W, respectively. Table S1: The diameters of colonies of untreated fungi (P0) and those subjected to five (P5), ten (P10), and fifteen (P15) treatments with a sublethal dose of DBD plasma measured after 3, 5, 7, and 14 days of incubation on PDA medium. Mean colony diameter values are presented with standard error for four replicates (*n* = 4). Additional information regarding one-way analysis of variance (ANOVA).

## Abbreviations

The following abbreviations are used in this manuscript:

DBD plasma	Dielectric barrier discharge plasma
MTT	3-(4,5-dimethylthiazol-2-yl)-2,5-diphenyl tetrazolium bromide
PDA PDB	Potato Dextrose Agar Potato Dextrose Broth
PCR	Polymerase Chain Reaction
F	Fungicide
