# Supplementary material for: Atmospheric Pressure Dielectric Barrier Discharge Plasma Treatment of Alternaria and Fusarium Species: Impact on Fungal Physiology, Antifungal Sensitivity, and Biofilm Formation
Source: Molecules. 2026 Jul 10;31(14):2422. doi: 10.3390/molecules31142422 (PMC13414195; doi:10.3390/molecules31142422)
Supplement: Supplementary file 1 [file molecules-31-02422-s001.zip › molecules-4362685-supplementary.pdf]

molecules

(Supplementary Information)

Atmospheric Pressure Dielectric Barrier Discharge Plasma Treatment of *Alternaria* and *Fusarium* Species: Impact on Fungal Physiology, Antifungal Sensitivity, and Biofilm Formation

Irena Maliszewska [1,*], Daria Nowinski [1] and Anna Baturo-Cieśniewska [2]

[1] Department of Organic and Medical Chemistry, Faculty of Chemistry, Wrocław University of Science and Technology, 50-371 Wrocław, Poland; daria.kocek@o2.pl
[2] Department of Microbiology and Plant Ecology, Faculty of Agriculture and Biotechnology, Bydgoszcz University of Science and Technology, 85-796 Bydgoszcz, Poland; anna.baturo-ciesnieska@pbs.edu.pl
* Correspondence: irena.helena.maliszewska@pwr.edu.pl

 https://doi.org/10.3390/molecules31142422

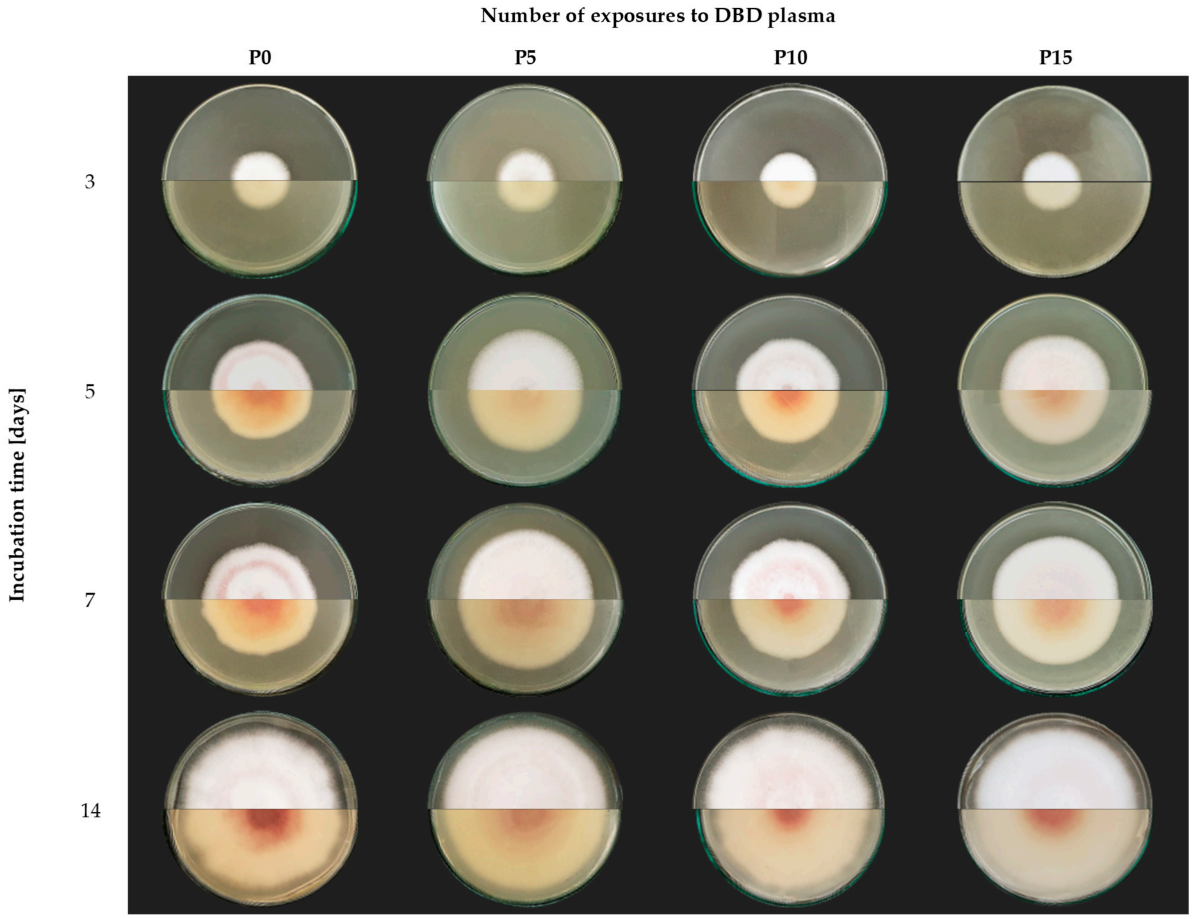

Figure S1. The morphology of *F. oxysporum* DSM 12646 colonies was assessed in untreated samples (P0) and after five (P5), ten (P10), and fifteen (P15) DBD plasma treatments on potato dextrose agar (PDA) over a 14-day culture period. The upper portion of each image displays the colony's obverse view, while the lower portion presents the reverse view. Images were captured after 3, 5, 7, and 14 days of incubation. Scale bar (3 cm) ⟷

https://doi.org/10.3390/molecules31142422

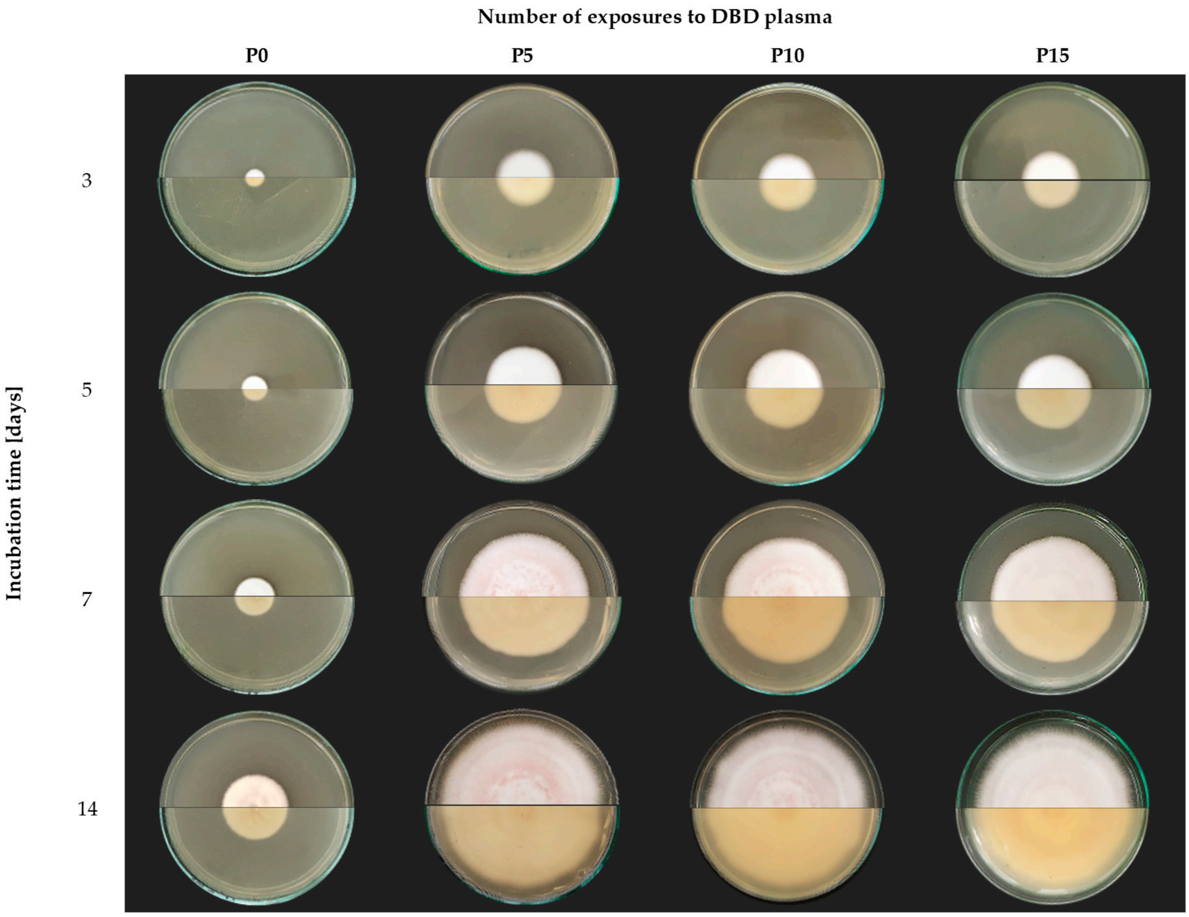

Figure S2. The morphology of *F. culmorum* DSM 1094 colonies was assessed in untreated samples (P0) and after five (P5), ten (P10), and fifteen (P15) DBD plasma treatments on potato dextrose agar (PDA) over a 14-day culture period. The upper portion of each image displays the colony's obverse view, while the lower portion presents the reverse view. Images were captured after 3, 5, 7, and 14 days of incubation. Scale bar (3 cm) ⟷

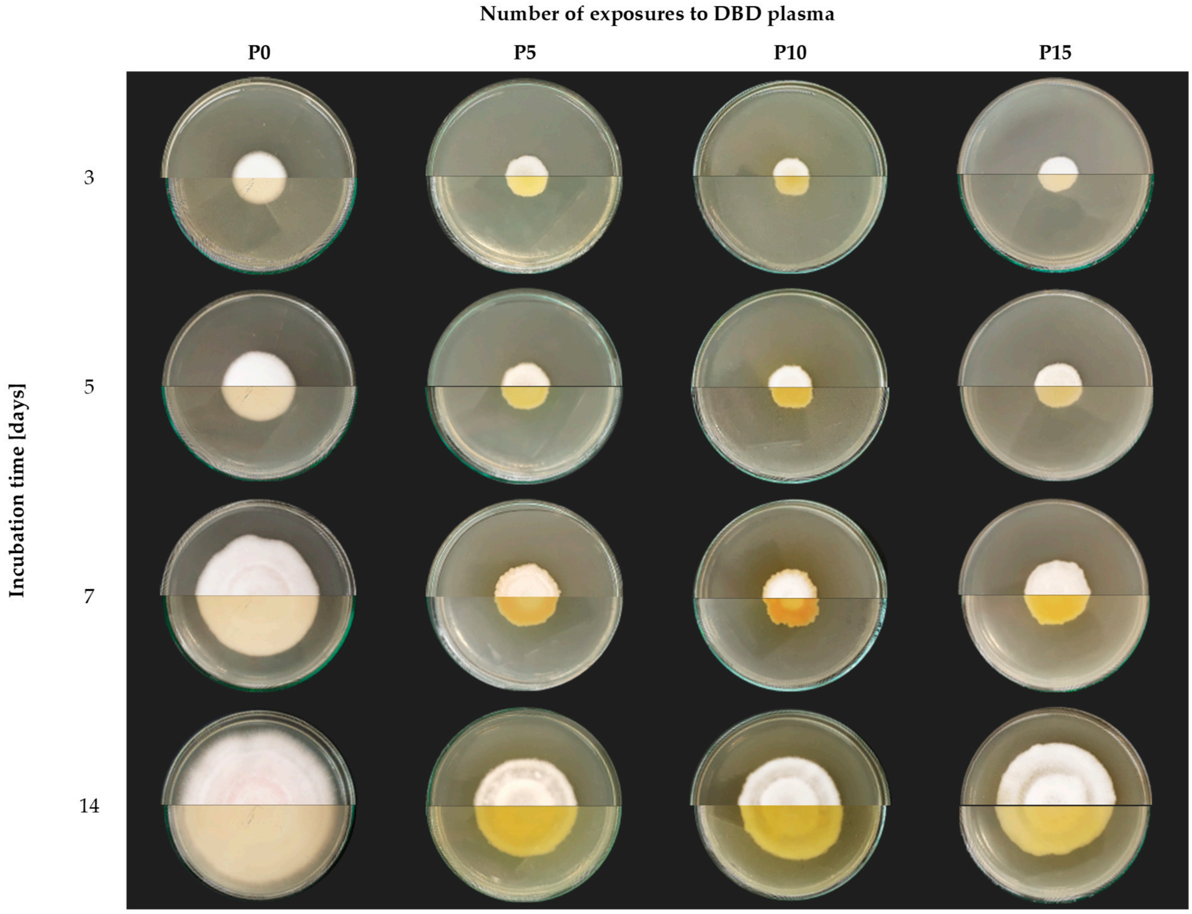

Figure S3. The morphology of *F. tricinctum* Ft11S-23 colonies was assessed in untreated samples (P0) and after five (P5), ten (P10), and fifteen (P15) DBD plasma treatments on potato dextrose agar (PDA) over a 14-day culture period. The upper portion of each image displays the colony's obverse view, while the lower portion presents the reverse view. Images were captured after 3, 5, 7, and 14 days of incubation. Scale bar (3 cm) ⟷

Table S1. The diameters of colonies of untreated fungi (P0) and those subjected to five (P5), ten (P10), and fifteen (P15) treatments with a sublethal dose of DBD plasma measured after 3, 5, 7, and 14 days of incubation on PDA medium. Mean colony diameter values are presented with standard error for four replicates (n=4).

**Strain**	**Incubation time [days]**	**Colony diameter [cm]**			
		**P0**	**P5**	**P10**	**P15**
A. alternata DSM 62010	3	1.1±0.1	1.0±0.0	1.0±0.1	1.1±0.1
	5	1.6±0.1[a]	1.6±0.1	1.6±0.1	2.0±0.1[a]
	7	2.0±0.1[b]	1.9±0.2[b]	2.0±0.1	2.4±0.1[b]
	14	3.3±0.1[c]	3.1±0.1[c]	3.3±0.1	3.5±0.1[c]
A. alternata Aa10S-23	3	2.2±0.1	2.2±0.1	2.4±0.1	2.1±0.1
	5	3.9±0.7	3.6±0.2	4.3±0.4	4.1±0.1
	7	5.0±0.8[d]	4.7±0.4[d]	5.1±0.1[d]	5.1±0.1[d]
	14	6.2±1.7[e]	6.9±0.3[e]	7.7±0.2[e]	7.9±0.1[e]
F. oxysporum DSM 12646	3	3.0±0.1[f]	3.0±0.1	2.8±0.1[f]	2.9±0.1[f]
	5	4.5±0.2	4.7±0.1	4.5±0.1	4.5±0.1
	7	5.9±0.1[g]	6.5±0.0[g]	5.9±0.2	6.4±0.1[g]
	14	7.9±0.2 [h]	7.8±0.1[h]	7.7±0.1[h]	7.8±0.1[h]
F. culmorum DSM 1094	3	1.0±0.1[i]	2.7±0.1[i]	2.7±0.0[i]	2.9±0.1[i]
	5	1.6±0.1[j]	5.1±0.1[j]	5.1±0.1[j]	5.4±0.2[j]
	7	2.2±0.1[k]	7.1±0.1[k]	7.0±0.0[k]	7.2±0.1[k]
	14	4.5±0.1	8.1±0.1	8.3±0.2	8.1±0.1
F. tricinctum Ft11S-23	3	2.7±0.1[l]	2.0±0.1[l]	2.0±0.1[l]	2.0±0.1[l]
	5	6.1±0.1[m]	3.0±0.1[m]	3.3±0.3[m]	3.3±0.1[m]
	7	7.4±0.2[n]	3.6±0.1[n]	4.0±0.3[n]	4.0±0.1[n]
	14	8.1±0.1[o]	5.4±0.2[o]	6.3±0.8[o]	6.0±0.1[o]

a, b, c, … – indicate significant differences according to Dunnett's post hoc test following one-way ANOVA ($p < 0.05$).

https://doi.org/10.3390/molecules31142422

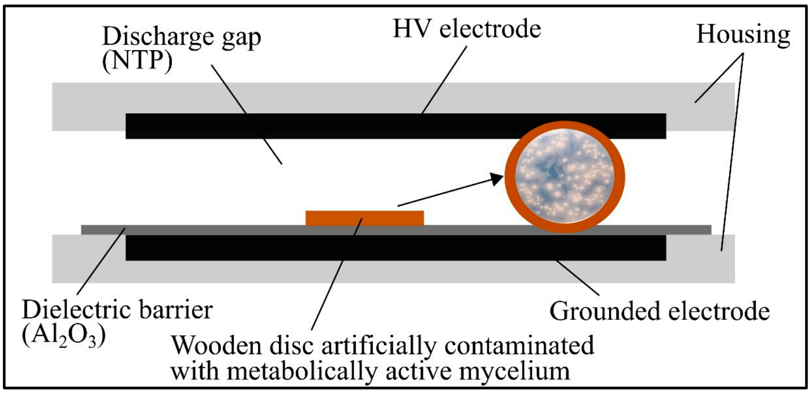

Figure S4. Schematic view of a plasma system using dielectric barrier discharges (DBD) in the air as the working gas, which was applied for multiple treatments of the studied plant pathogens. The plasma reactor was powered by a high-voltage pulse generator with a modulated frequency (Dora PS, Wroclaw, Poland). The pulse amplitude, frequency, and discharge power were 5.5 kV, 38 kHz, and 9 W, respectively.

Additional information regarding one-way analysis of variance (ANOVA).

One-way ANOVA

		Sum of Squares	df	Mean Square	F	Sig.
A2_alternataDSM	Between Groups	25.027	3	8.342	8898.505	<.001
	Within Groups	.019	20	.001		
	Total	25.046	23			
A2_alternataAa	Between Groups	17.967	3	5.989	691.357	<.001
	Within Groups	.173	20	.009		
	Total	18.140	23			
A2_Oxysporum	Between Groups	5.763	3	1.921	20.700	<.001
	Within Groups	1.856	20	.093		
	Total	7.619	23			
A2_Culmorum	Between Groups	72.556	3	24.185	4524.832	<.001
	Within Groups	.107	20	.005		
	Total	72.663	23			
A2_Tricinctum	Between Groups	.222	3	.074	8.753	<.001
	Within Groups	.169	20	.008		
	Total	.392	23			

Multiple Comparisons

Dunnett t (2-sided)[a]

Dependent Variable	(I) P_group	(J) P_group	Mean Difference (I-J)	Std. Error	Sig.	95% Confidence Interval	
						Lower Bound	Upper Bound
A2_alternataDSM	5.00	.00	-2.74167*	.01768	<.001	-2.7866	-2.6968
	10.00	.00	-2.03167*	.01768	<.001	-2.0766	-1.9868
	15.00	.00	-1.18833*	.01768	<.001	-1.2332	-1.1434
A2_alternataAa	5.00	.00	-1.78833*	.05374	<.001	-1.9248	-1.6518
	10.00	.00	-2.30667*	.05374	<.001	-2.4432	-2.1702
	15.00	.00	-1.66000*	.05374	<.001	-1.7965	-1.5235
A2_Oxysporum	5.00	.00	.63167*	.17588	.005	.1849	1.0785
	10.00	.00	-.20833	.17588	.511	-.6551	.2385
	15.00	.00	-.73667*	.17588	.001	-1.1835	-.2899
A2_Culmorum	5.00	.00	1.91000*	.04221	<.001	1.8028	2.0172
	10.00	.00	3.53833*	.04221	<.001	3.4311	3.6456
	15.00	.00	4.60167*	.04221	<.001	4.4944	4.7089
A2_Tricinctum	5.00	.00	-.02000	.05313	.965	-.1550	.1150
	10.00	.00	.19833*	.05313	.004	.0634	.3333
	15.00	.00	-.04333	.05313	.755	-.1783	.0916

*. The mean difference is significant at the 0.05 level.

a. Dunnett t-tests treat one group as a control. and compare all other groups against it.

ANOVA

		Sum of Squares	df	Mean Square	F	Sig.
A3_alternataDSM	Between Groups	886.458	3	295.486	53.970	<.001
	Within Groups	109.500	20	5.475		
	Total	995.958	23			
A3_alternataAa	Between Groups	794.167	3	264.722	52.077	<.001
	Within Groups	101.667	20	5.083		
	Total	895.833	23			
A3_Oxysporum	Between Groups	725.000	3	241.667	105.455	<.001
	Within Groups	45.833	20	2.292		
	Total	770.833	23			
A3_Tricinctum	Between Groups	1385.917	3	461.972	231.468	<.001
	Within Groups	39.917	20	1.996		
	Total	1425.833	23			

https://doi.org/10.3390/molecules31142422

ANOVA F. Culmorum

A3_Fusarium

	Sum of Squares	df	Mean Square	F	Sig.
Between Groups	813,198	3	271,066	167,886	<,001
Within Groups	32,292	20	1,615		
Total	845,490	23			

Multiple Comparisons

Dunnett t (2-sided)[a]

Dependent Variable	(I) P_grupujaca	(J) P_grupujaca	Mean Difference (I-J)	Std. Error	Sig.	95% Confidence Interval Lower Bound	Upper Bound
A3_alternataDSM	5.00	.00	2.66667	1.35093	.150	-.7652	6.0985
	10.00	.00	11.33333*	1.35093	<.001	7.9015	14.7652
	15.00	.00	14.83333*	1.35093	<.001	11.4015	18.2652
A3_alternataAa	5.00	.00	14.83333*	1.30171	<.001	11.5265	18.1402
	10.00	.00	13.00000*	1.30171	<.001	9.6932	16.3068
	15.00	.00	7.83333*	1.30171	<.001	4.5265	11.1402
A3_Oxysporum	5.00	.00	-3.50000*	.87401	.002	-5.7203	-1.2797
	10.00	.00	3.50000*	.87401	.002	1.2797	5.7203
	15.00	.00	11.33333*	.87401	<.001	9.1130	13.5536
A3_Tricinctum	5.00	.00	5.50000*	.81565	<.001	3.4280	7.5720
	10.00	.00	-7.58333*	.81565	<.001	-9.6554	-5.5113
	15.00	.00	-14.58333*	.81565	<.001	-16.6554	-12.5113

*. The mean difference is significant at the 0.05 level.

a. Dunnett t-tests treat one group as a control. and compare all other groups against it.

Multiple Comparisons F. Culmorum

Dependent Variable: A3_Fusarium

Dunnett t (2-sided)[a]

(I) P_grupujaca	(J) P_grupujaca	Mean Difference (I-J)	Std. Error	Sig.	95% Confidence Interval Lower Bound	Upper Bound
5,00	,00	4,75000*	,73362	<,001	2,8863	6,6137
10,00	,00	15,41667*	,73362	<,001	13,5530	17,2803
15,00	,00	2,91667*	,73362	,002	1,0530	4,7803

*. The mean difference is significant at the 0.05 level.

a. Dunnett t-tests treat one group as a control, and compare all other groups against it.

ANOVA Alternata DSM

		Sum of Squares	df	Mean Square	F	Sig.
A6_alternataDSM5	Between Groups	.480	1	.480	4.3269E+31	<.001
	Within Groups	.000	10	.000		
	Total	.480	11			
A6_alternataDSM7	Between Groups	,645	3	,215	860,000	<.001
	Within Groups	,005	20	,000		
	Total	,650	23			
A6_alternataDSM14	Between Groups	.480	2	.240	1.082E+31	<.001
	Within Groups	.000	15	.000		
	Total	.480	17			

Multiple Comparisons Alternata DSM

Dunnett t (2-sided)[a]

Dependent Variable	(I) P_grupuhaca	(H) P_grupuhaca	Mean Difference (I-H)	Sig.	95% Confidence Interval Lower Bound	Upper Bound
A6_alternataDSM5	5.00	.00	.00000	1.000	.0000	.0000
	10.00	.00	.00000	1.000	.0000	.0000
	15.00	.00	.40000*	<.001	.4000	.4000
A6_alternataDSM7	5.00	.00	-.10000*	<.001	-.1000	-.1000
	10.00	.00	.00000	1.000	.0000	.0000
	15.00	.00	.40000*	<.001	.4000	.4000
A6_alternataDSM14	5.00	.00	-.20000*	<.001	-.2000	-.2000
	10.00	.00	.00000	1.000	.0000	.0000
	15.00	.00	.20000*	<.001	.2000	.2000

*. The mean difference is significant at the 0.05 level.

a. Dunnett t-tests treat one group as a control. and compare all other groups against it.

ANOVA Alternata Aa

		Sum of Squares	df	Mean Square	F	Sig.
A6_alternataAa3	Between Groups	.285	3	.095	.	.
	Within Groups	.000	20	.000		
	Total	.285	23			
A6_alternataAa5	Between Groups	1.605	3	.535	.	.
	Within Groups	.000	20	.000		
	Total	1.605	23			
A6_alternataAa7	Between Groups	1.845	3	.615	$6.93E + 30$	<.001
	Within Groups	.000	20	.000		
	Total	1.845	23			

https://doi.org/10.3390/molecules31142422

A6_alternataAa14	Between Groups	10.965	3	3.655	6.17E+31	<.001
	Within Groups	.000	20	.000		
	Total	10.965	23			

Multiple Comparisons Alternata Aa

Dunnett t (2-sided)[a]

Dependent Variable	(I) P_grupuhaca	(H) P_grupuhaca	Mean Difference (I-H)	Sig.	95% Confidence Interval	
					Lower Bound	Upper Bound
A6_alternataAa7	5.00	.00	-,30000	<.001	-,3232	-,2768
	10.00	.00	,10000	<.001	,0768	,1232
	15.00	.00	,10000	<.001	,0768	,1232
A6_alternataAa14	5.00	.00	.70000*	<.001	.7000	.7000
	10.00	.00	1.50000*	<.001	1.5000	1.5000
	15.00	.00	1.70000*	<.001	1.7000	1.7000

*. The mean difference is significant at the 0.05 level.

a. Dunnett t-tests treat one group as a control. and compare all other groups against it.

ANOVA Oxysporium

		Sum of Squares	df	Mean Square	F	Sig.
A6_oxysporum3	Between Groups	.165	3	.055	7.436E+30	<.001
	Within Groups	.000	20	.000		
	Total	.165	23			
A6_oxysporum5	Between Groups	.180	3	.060	.	.
	Within Groups	.000	20	.000		
	Total	.180	23			
A6_oxysporum7	Between Groups	1.845	3	.615	6.929E+30	<.001
	Within Groups	.000	20	.000		
	Total	1.845	23			
A6_oxysporum14	Between Groups	.120	3	.040	1.352E+30	<.001
	Within Groups	.000	20	.000		
	Total	.120	23			

Multiple Comparisons Oxysporium

Dunnett t (2-sided)[a]

Dependent Variable	(I) P_grupuhaca	(H) P_grupuhaca	Mean Difference (I-H)	Sig.	95% Confidence Interval	
					Lower Bound	Upper Bound
A6_oxysporum3	5.00	.00	.00000	1.000	.0000	.0000
	10.00	.00	-.20000*	<.001	-.2000	-.2000

	15.00	.00	-.10000*	<.001	-.1000	-.1000
A6_oxysporum7	5.00	.00	.60000*	<.001	.6000	.6000
	10.00	.00	.00000	1.000	.0000	.0000
	15.00	.00	.50000*	<.001	.5000	.5000
A6_oxysporum14	5.00	.00	-.10000*	<.001	-.1000	-.1000
	10.00	.00	-.20000*	<.001	-.2000	-.2000
	15.00	.00	-.10000*	<.001	-.1000	-.1000

*. The mean difference is significant at the 0.05 level.

a. Dunnett t-tests treat one group as a control. and compare all other groups against it.

ANOVA Culmorum

		Sum of Squares	df	Mean Square	F	Sig.
A6_culmorum3	Between Groups	14.205	3	4.735	3.201E+32	<.001
	Within Groups	.000	20	.000		
	Total	14.205	23			
A6_culmorum5	Between Groups	58.680	3	19.560	6.223E+32	<.001
	Within Groups	.000	20	.000		
	Total	58.680	23			
A6_culmorum7	Between Groups	108.165	3	36.055	1.219E+33	<.001
	Within Groups	.000	20	.000		
	Total	108.165	23			
A6_culmorum14	Between Groups	60.660	3	20.220	.	.
	Within Groups	.000	20	.000		
	Total	60.660	23			

Multiple Comparisons Culmorum

Dunnett t (2-sided)[a]

Dependent Variable	(I) P_grupuhaca	(H) P_grupuhaca	Mean Difference (I-H)	Sig.	95% Confidence Interval	
					Lower Bound	Upper Bound
A6_culmorum3	5.00	.00	1.70000*	<.001	1.7000	1.7000
	10.00	.00	1.70000*	<.001	1.7000	1.7000
	15.00	.00	1.90000*	<.001	1.9000	1.9000
A6_culmorum5	5.00	.00	3.50000*	<.001	3.5000	3.5000
	10.00	.00	3.50000*	<.001	3.5000	3.5000
	15.00	.00	3.80000*	<.001	3.8000	3.8000
A6_culmorum7	5.00	.00	4.90000*	<.001	4.9000	4.9000
	10.00	.00	4.80000*	<.001	4.8000	4.8000
	15.00	.00	5.00000*	<.001	5.0000	5.0000

*. The mean difference is significant at the 0.05 level.

a. Dunnett t-tests treat one group as a control. and compare all other groups against it.

https://doi.org/10.3390/molecules31142422

ANOVA Tricintum

		Sum of Squares	df	Mean Square	F	Sig.
A6_tricintum3	Between Groups	2.205	3	.735	9.93E+31	<.001
	Within Groups	.000	20	.000		
	Total	2.205	23			
A6_tricintum5	Between Groups	38.205	3	12.735	2.870E+32	<.001
	Within Groups	.000	20	.000		
	Total	38.205	23			
A6_tricintum7	Between Groups	56.820	3	18.940	6.402E+32	<.001
	Within Groups	.000	20	.000		
	Total	56.820	23			
A6_tricintum14	Between Groups	24.300	3	8.100	2.738E+32	<.001
	Within Groups	.000	20	.000		
	Total	24.300	23			

Multiple Comparisons Tricintum

Dunnett t (2-sided)[a]

Dependent Variable	(I) P_grupuhaca	(H) P_grupuhaca	Mean Difference (I-H)	Sig.	95% Confidence Interval	
					Lower Bound	Upper Bound
A6_tricintum3	5.00	.00	-.70000*	<.001	-.7000	-.7000
	10.00	.00	-.70000*	<.001	-.7000	-.7000
	15.00	.00	-.70000*	<.001	-.7000	-.7000
A6_tricintum5	5.00	.00	-3.10000*	<.001	-3.1000	-3.1000
	10.00	.00	-2.80000*	<.001	-2.8000	-2.8000
	15.00	.00	-2.80000*	<.001	-2.8000	-2.8000
A6_tricintum7	5.00	.00	-3.80000*	<.001	-3.8000	-3.8000
	10.00	.00	-3.40000*	<.001	-3.4000	-3.4000
	15.00	.00	-3.40000*	<.001	-3.4000	-3.4000
A6_tricintum14	5.00	.00	-2.70000*	<.001	-2.7000	-2.7000
	10.00	.00	-1.80000*	<.001	-1.8000	-1.8000
	15.00	.00	-2.10000*	<.001	-2.1000	-2.1000

*. The mean difference is significant at the 0.05 level.

a. Dunnett t-tests treat one group as a control. and compare all other groups against it.

Disclaimer/Publisher's Note: The statements, opinions and data contained in all publications are solely those of the individual author(s) and contributor(s) and not of MDPI and/or the editor(s). MDPI and/or the editor(s) disclaim responsibility for any injury to people or property resulting from any ideas, methods, instructions or products referred to in the content.
